# Immune Checkpoint Inhibitors in Non-Small Cell Lung Cancer: Progress, Challenges, and Prospects

**DOI:** 10.3390/cells11030320

**Published:** 2022-01-19

**Authors:** Shengjie Tang, Chao Qin, Haiyang Hu, Tao Liu, Yiwei He, Haiyang Guo, Hang Yan, Jun Zhang, Shoujun Tang, Haining Zhou

**Affiliations:** 1 Department of Thoracic Surgery, Suining Central Hospital, An Affiliated Hospital of Chongqing Medical University, Suining 629099, China; tsj12345tsj@gmail.com (S.T.); snchqc@gmail.com (C.Q.); wujieyang19941010@gmail.com (H.H.); liutao521618@gmail.com (T.L.); heyiwei1232021@gmail.com (Y.H.); haiyangguo@stu.cdutcm.edu.cn (H.G.); 15808588088yh@gmail.com (H.Y.); zhangjun202103@gmail.com (J.Z.); 2 Institute of Surgery, Graduate School, Zunyi Medical University, Zunyi 563002, China; 3 Institute of Surgery, Graduate School, Chengdu University of TCM, Chengdu 610075, China

**Keywords:** non-small cell lung cancer, immune checkpoint inhibitors, PD-1/PD-L1, CTLA-4, immunotherapy, biomarkers

## Abstract

Non-small cell lung cancer is one of the most common types of malignances worldwide and the main cause of cancer-related deaths. Current treatment for NSCLC is based on surgical resection, chemotherapy, radiotherapy, and targeted therapy, with poor therapeutic effectiveness. In recent years, immune checkpoint inhibitors have applied in NSCLC treatment. A large number of experimental studies have shown that immune checkpoint inhibitors are safer and more effective than traditional therapeutic modalities and have allowed for the development of better guidance in the clinical treatment of advanced NSCLC patients. In this review, we describe clinical trials using ICI immunotherapies for NSCLC treatment, the available data on clinical efficacy, and the emerging evidence regarding biomarkers.

## 1. Non-Small Cell Lung Cancer

**Figure 1:**
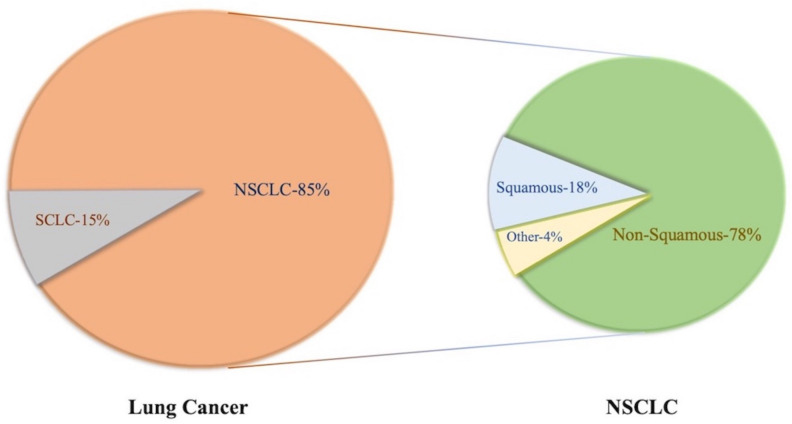
Pathological classification of lung cancer and its corresponding prevalence.

Lung cancer is one of the most common malignances—annual morbidity and mortality rates are still rising, and patient is often diagnosed at advanced stage, that the reason for the poor progression as treatment options are limited. The International Agency for Research on Cancer reported that the five-year survival rate of patients with lung cancers was only 17.7% [1]. With regard to the pathological types, lung cancer can be divided into two main subtypes: small cell lung cancer (SCLC) and non-small cell lung cancer (NSCLC). NSCLC is further divided into several subgroups, including squamous cell carcinoma, adenocarcinoma large cell carcinoma, and sarcomatoid neoplasms (Figure 1). Since 2004, lung adenocarcinoma has become the most common histological cancer type diagnosed worldwide [2], despite the incidence of lung cancer varying across different countries, regions, and races [3]. Epidemiological studies have found that squamous cell carcinoma is more common in men while adenocarcinoma is more common in women [4]. Therefore, it is important to know the risk factors and mechanism of NSCLC progression and explore better treatment options for NSCLC.

There are many risk factors in lung cancers, such as smoking, air pollution, radon and asbestos, occupational exposure, hereditary susceptibility, hormonal and viral factors, aging, radiation, and diet [5]. Smoking is considered as the highest risk associated with lung cancer developing. Not only SCLC and squamous cell lung cancer are closely related to smoking, increasing evidence shows that adenocarcinoma is also closely related to smoking [6]. In addition, Lung cancer is related to aging to some extent. However, no single risk factor appears to be dominant [7]. The diversity of risk factors suggests that people should be able to address multiple aspects in preventing lung cancer.

## 2. Tumor Immune Escape

**Figure 2:**
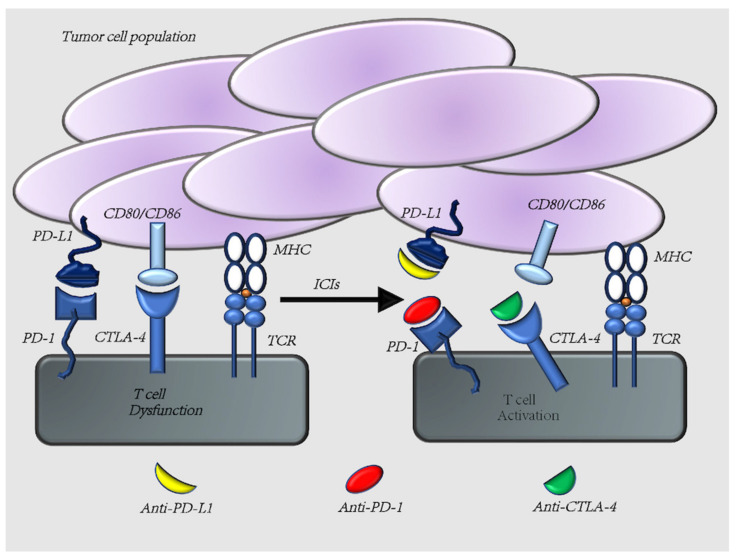
Tumor immune mechanism. T cells express PD-1 and CTLA-4 on their surface. Interaction with its ligands, PD-L1 and CD80/CD86, respectively, results in negative regulation of T cells. Therefore, anti-PD-1/PD-L1 and anti-CTLA-4 pathway antibodies would induce an upregulation of T cell activity.

The growth and spread of malignant tumors are a complex process that depends not only on the characteristics of the tumor cells but is also influenced by the immune system [8], and the occurring of immune escape is a very important step for tumorigenesis. Tumor immune escape means tumor cells can evade immune surveillance by reprogramming the biology of immune cells, a process also known as immune editing. The mechanism of tumor immune escape is complex, including the involvement of multiple cells and molecules. Among them, immune checkpoints PD-1/PD-L1, CTLA-4 play an important role. The nature of PD-1/PD-L1, CTLA-4 is a ligand or receptor for the interaction between tumor cells and immune cells, which serves as immunomodulatory role (Figure 2).

CTLA-4 is highly homologous to CD28 and its ligand is also CD80/CD86. The affinity of CTLA-4 to CD80/CD86 is 20 times higher than that of CD28 to CD80/CD86. Apparently, CTLA-4 can compete with CD28 for binding to CD80/CD86 and is more dominant. Generally, CTLA-4 is expressed after T cell activation and its effect, so its role is to downregulate or stop T cell activation [9]. The main reason is that the cytoplasmic region of CTLA-4 includes an immunoreceptor tyrosine-based inhibitory motif (ITIM) that transmits inhibitory signals. This effect weakens the immune response and contributes to the escape of tumor cells.

PD-1, which ligands are PD-L1 and PD-L2, is expressed on the surface of T cells shortly after their activation [10]. Binding of PD-1 on the surface of T cells to PD-L1 on tumor cells inhibits T cell-associated kinases and prevents the development of cytotoxic T cell responses to tumors. Thus, T cells cannot identify and kill tumor cells, leading to the failure of cytotoxic T lymphocytes (CTLs) and causing immune avoidance [11,12]. In addition, PD-1 binding with its ligand can inhibit the proliferation of T lymphocytes and the production of cytokines such as IL-2 and IFN-Y, as well as inhibit the proliferation and differentiation of B lymphocytes and the secretion of Ig, thus weakening the immune effect of effector cells. PD-L1 is also expressed in normal tissues, but its expression is limited. High expression of PD-L1 results in immune escape, as in lung cancer, where PD-L1 is expressed in 35–95% of NSCLC patients [13]. Studies have shown that the expression of PD-L1 in NSCLC is associated with shorter survival and poor prognosis of patients [14]. Meanwhile, high expression of PD-L1 in lung cancer tissues is often accompanied by high levels of T cell infiltration, which often means depletion of T cells and their antitumor effects. This is the strategy used by lung cancer cells to avoid detection by immune system surveillance and, thus, elimination [15]. Overall, the immune checkpoints PD-1/PD-L1 and CTLA-4 often weaken the efficacy of immune cells when modulating tumor immunity, ultimately leading to tumor cell escape.

In recent years, despite the increasing number of treatment strategies for lung cancer, almost no treatment option has limitations. For example, the more emerging targeted agents, such as KRAS, HER2, and NTRK as targets for NSCLC, but resistance to targeted agents in patients with advanced NSCLC remains a challenge [16]. Fortunately, the immune checkpoints PD-1/PD-L1 and CTLA-4 provide a new and promising therapeutic strategy for the treatment of NSCLC. In the following sections, we discuss the clinical efficacy, safety, therapeutic strategies, challenges, and expectations of drugs related to the treatment of NSCLC with PD-1/PD-L1 and CTLA-4 as targets.

## 3. ICIs Monotherapy

The immune system appears to have both tumor-suppressive and antitumor effects, which depends on the interaction between the tumor microenvironment and the immune system. Tumor immunotherapy is mainly based on enhancing the recognition of tumor antigens by antigen-presenting cells (APCs) and T lymphocytes, enhancing the immune response, relieving the inhibitory effect of immune cells, and promoting the antitumor immune response [17]. ICIs (immune checkpoint inhibitors) are committed to relieving the inhibitory effect of tumors on T lymphocytes, thereby enhancing the activation, proliferation, and differentiation of T lymphocytes and enhancing immune function, increasing the abundance of proteins involved in the immune response. ICIs have certain advantages in terms of efficacy and safety and bring new hope for NSCLC treatment [18]. In the next section, we discuss the current status of monotherapy or combination therapy based on ICIs for the treatment of NSCLC, and we summarized important clinical trials in the field of immune monotherapy.

### 3.1. Nivolumab

Nivolumab is an all-human IgG4 monoclonal antibody that targets PD-1. Nivolumab inhibits the expression of PD-1 receptor on activated T cells by eliminating the inhibitory signal, and the number of effector T cells is increased via induction or expansion [19]. Nivolumab is the first humanized monoclonal antibody against the PD-1 receptor approved for marketing by the FDA, and it is also the first antibody in this class to undergo NSCLC-related clinical trials. In July 2014, it was approved by the regulatory agency in Japan for the treatment of unresectable melanoma. This was the first time that a PD-1 inhibitor was approved for clinical use across the world [20].

In a randomized, open-label, international phase III study (CheckMate 057) [21], researchers assigned patients with non-squamous non-small cell lung cancer (NS-NSCLC) that had progressed during or after platinum-based doublet chemotherapy to receive nivolumab at a dose of 3 mg/kg of body weight every two weeks or docetaxel at a dose of 75 mg/m^2^ of body-surface area every three weeks. The results indicated that OS was longer in the nivolumab group than in the docetaxel group. Nivolumab had a higher one-year PFS rate than docetaxel. Grade 3 or 4 treatment-related adverse events occurred in 10% of patients in the nivolumab group, compared to 54% in the docetaxel group. The experimental results suggest that in patients with advanced NS-NSCLC who progressed during or after platinum chemotherapy, the nivolumab group had a longer OS and a better safety profile than the docetaxel group.

Similarly, another study evaluated the efficacy and safety of nivolumab versus docetaxel in the patient population (CheckMate 017) [22]. The results showed that, after patients with squamous advanced NSCLC had received preliminary treatment, regardless of the PD-L1 expression level, the OS, response rate, and PFS were significantly increased in the nivolumab group compared with the docetaxel group. Specifically, the median OS was 9.2 months with nivolumab versus 6.0 months with docetaxel, the risk of death was 41% lower with nivolumab than with docetaxel.

Furthermore, another study showed that nivolumab was not significantly associated with increased PFS (4.2 vs. 5.9 months) compared to chemotherapy in patients with previously untreated stage IV or recurrent NSCLC with 5% or more PD-L1 expression. OS was similar between the two groups (14.4 vs. 13.2 months). However, nivolumab has a good safety profile compared to chemotherapy [23].

A pooled analysis of CheckMate 017 and CheckMate 057 studies showed that the two-year OS rates with nivolumab versus docetaxel were 23% versus 8% in squamous NSCLC and 29% versus 16% in NS-NSCLC [24]. Sustained responses were observed with nivolumab; 10 (37%) of 27 confirmed responders with squamous NSCLC and 19 (34%) of 56 with NS-NSCLC had ongoing responses at the two-year follow-up. No patient in either docetaxel group had an ongoing response. In the pooled analysis, the risk of death with nivolumab versus docetaxel was lower by 28%, and the rates of treatment-related adverse events were lower with nivolumab than with docetaxel.

Nivolumab is undoubtedly pioneering in the research of NSCLC immune monotherapy. These data, when compared to those of previously treated patients with advanced NSCLC, suggest that nivolumab has long-term clinical benefits and is well tolerated. Therefore, nivolumab was proven to be effective and safe in patients with advanced NSCLC.

### 3.2. Pembrolizumab

Pembrolizumab is a humanized monoclonal IgG4-κ isotype antibody that binds to the PD-1 receptor, preventing interaction with PD-L1 and PD-L2.

KEYNOTE-001 served as the prelude to the series of pembrolizumab studies. This study enrolled 101 treatment-naive and 449 previously treated patients, and the median follow-up was 60.6 months. Up to the point data cutoff on 5 November 2018, 450 patients (82%) had died, and the median OS was 22.3 months in treatment-naive patients and 10.5 months in previously treated patients. The estimated five-year OS was 23.2% for treatment-naive patients and 15.5% for previously treated patients [25]. Pembrolizumab monotherapy provided durable antitumor activity and high five-year OS rates in treatment-naive patients or patients previously treated for advanced NSCLC. Notably, the five-year OS rate exceeded 25% among patients with a PD-L1 tumor proportion score of 50% or greater. Pembrolizumab demonstrated a tolerable long-term safety profile with little evidence of late onset or new toxicity.

After KEYNOTE-001, a new study rewrote the NSCLC treatment landscape. KEYNOTE-024 is the first phase III study to report the five-year efficacy of first-line immunotherapy for advanced NSCLC. This study recently achieved breakthrough progress; after five years of follow-up, it determined that pembrolizumab is an effective first-line treatment for patients with PD-L1 TPS ≥ 50% of advanced NSCLC [26]. The results of the KEYNOTE-024 study showed that in patients with PD-L1 TPS ≥ 50%, pembrolizumab first-line monotherapy significantly improved patients PFS and reduced their disease progression by 50% as well as significantly improved the objective response rate (ORR) (44.8% vs. 27.8%) compared to chemotherapy. Based on the results of the KEYNOTE-024 study, the FDA approved use of pembrolizumab for newly diagnosed advanced NSCLC patients with a ≥50% PD-L1 expression rate and no clear driver gene mutation. KEYNOTE-024 is the first monotherapy study demonstrating the improved OS of patients with advanced NSCLC. In the past, two-drug chemotherapy regimens or even three-drug combination regimens combined with bevacizumab were used for this population of patients, but they failed to achieve a boost in OS similar to that of the KEYNOTE-024 study.

Based on the research of KEYNOTE-024, KEYNOTE-042 further expanded upon the population deemed to benefit from pembrolizumab monotherapy [27]. In KEYNOTE-042, a randomized, open-label, controlled, phase III trial, 1274 patients with a PD-L1 TPS of 1% or greater were enrolled and either allocated to receive pembrolizumab (*n* = 637) or chemotherapy (*n* = 637), and included in addition to an intention-to-treat population. Of the patients, 599 (47%) had a TPS of 50% or greater and 818 patients (64%) had a TPS of 20% or greater. As of 26 February 2018, the median follow-up was 12.8 months. OS was significantly longer in the pembrolizumab group than in the chemotherapy group in all three TPS populations. This research shows that pembrolizumab monotherapy can be extended as a first-line therapy to patients with locally advanced or metastatic non-small cell lung cancer without sensitizing EGFR or ALK alterations and with low PD-L1 TPS. 

Although the above studies have confirmed that people with high PD-L1 expression can benefit from immune monotherapy, this group of patients accounts for a low percentage of NSCLC, and how to expand the population benefiting from immunotherapy and obtain longer survival will be the focus of future research.

### 3.3. Atezolizumab

Atezolizumab is a humanized IgG1 monoclonal antibody targeting PD-L1 that inhibits PD-L1 and PD-L1 and B7-1 interactions. Atezolizumab has no antibody-dependent cell-mediated cytotoxicity (ADCC) and can theoretically avoid killing T cells directly activated by tumors.

In an open phase II trial (POPLAR), 287 patients with advanced NSCLC who had progressed after platinum-based chemotherapy were divided into two groups that received either atezolizumab or docetaxel [28]. Patients receiving platinum-based chemotherapy and atezolizumab had significantly improved OS, with a median of 12.6 months compared to 9.7 months in NSCLC, and higher PD-L1 tumor cell expression as well as higher tumor filtration cell expression observed in in patients receiving docetaxel.

In addition, a randomized, open-label, phase III trial (OAK) evaluated the efficacy and safety of atezolizumab versus docetaxel in previously treated NSCLC patients [29]. The median OS was 13.8 months in the atezolizumab group and 9.6 months in the docetaxel group, respectively, and the improvement in median OS was more significant in strong PD-L1-positive patients, suggesting that PD-L1 expression can effectively predict the effect of anti-PD-L1 treatment. Fewer patients had treatment-related grade 3 or 4 adverse events with atezolizumab versus docetaxel. The results demonstrate that atezolizumab therapy led to clinically relevant improvements in OS regardless of PD-L1 expression or histology, with a good safety profile.

In May 2016, based on the POPLAR and OAK studies, atezolizumab was approved by the FDA for use in NSCLC after platinum-based chemotherapy has progressed.

Almost all clinical studies of drugs are pushed from the second line to the first line, and atezolizumab is no exception. IMpower110 evaluated the efficacy and safety of atezolizumab as a first-line therapy in NSCLC patients with PD-L1 expression [30]. This study involved patients with non-squamous or squamous NSCLC who had not previously received chemotherapy. The results showed that the median OS of the atezolizumab group was 7.1 months longer than that of chemotherapy (20.2 vs. 13.1 months). Adverse events occurred in 90.2% of patients in the atezolizumab group and 94.7% in the chemotherapy group among all patients who could be assessed for safety. Atezolizumab was shown to result in a significantly longer OS than platinum-based chemotherapy in NSCLC patients with high PD-L1 expression, regardless of histological type.

In May 2020, the FDA approved atezolizumab monotherapy as a first-line treatment for patients with metastatic NSCLC with EGFR/ALK-negative and high PD-L1 expression.

### 3.4. Avelumab

Avelumab is a human Ig-G1 monoclonal antibody that targets PD-L1.

The safety of and tolerability for avelumab in expressing its antitumor activity were demonstrated based on its use in first-line treatment of patients with advanced NSCLC [31]. In this study, 10 mg/kg avelumab was intravenously administered every two weeks to patients with untreated, metastatic, or relapsed NSCLC. The objective response rate was 19.9%, including a complete response in three and a partial response in 28 cases. The median PFS was 4.0 months, while the six-month PFS rate was 38.5%. Meanwhile, the median OS was 14.1 months and the 12-month OS rate was 56.6%. Immune-related adverse events and infusion-related reactions occurred in 31 (19.9%) and 40 (25.6%) patients, respectively.

Recently, Keunchil et al. reported the two-year follow-up results from the JAVELIN Lung 200 phase III trial [32]. In 792 patients, 529 had PD-L1-positive tumors. As of 4 March 2019, the median duration of follow-up for OS in the PD-L1+ population was 35.4 months in the avelumab arm and 34.7 months in the docetaxel arm. In the PD-L1+ population, the two-year OS rates with avelumab versus docetaxel were 29.9% and 20.5%, respectively; in the population with greater than or equal to 50% PD-L1+ expression, the two-year OS rates were 36.4% versus 17.7%, while in the subgroup with greater than or equal to 80% expression were 40.2% versus 20.3%, respectively. The safety profiles for both arms were consistent with the primary analysis.

Although the JAVELIN Lung 200 primary analysis revealed that avelumab did not significantly prolong OS versus docetaxel in patients with platinum-treated PD-L1 positive NSCLC, post-hoc analyses after two years of follow-up revealed that the two-year OS rates were doubled with avelumab in subgroups with higher PD-L1 expression. Although the JAVELIN study did not achieve the expected results, the latest two-year follow-up results indicate the potential of avelumab in NSCLC immunotherapy.

### 3.5. Durvalumab

Durvalumab is a humanized IgG1κ monoclonal antibody that targets PD-L1 with high affinity and selectively blocks PD-L1 binding to PD-1.

In a study of OS associated with durvalumab treatment after chemoradiotherapy in stage III NSCLC (PACIFIC study) [33], 713 patients were enrolled. The results indicate that the 24-month OS rate was 66.3% in the durvalumab group, compared to 55.6% in the placebo group. Durvalumab significantly prolonged the OS, compared to the placebo. This trial reconfirms that durvalumab consolidation therapy after deterministic chemoradiotherapy (CRT) improves overall and progression-free survival (PFS).

The applicable population of the PACIFIC study is non-resectable stage III NSCLC, pioneering the immunotherapy of stage III NSCLC. In February 2018, the FDA announced the approval of durvalumab for patients with stage III NSCLC whose tumors cannot be removed by surgery but whose condition has not progressed under the existing chemotherapy and radiotherapy protocols.

Recently, Corinne et al. report updated analyses of OS and PFS, approximately four years after the last patient was randomized. As of 20 March 2020, updated OS and PFS remained consistent with the primary analyses. The median OS for durvalumab was reached (47.5 months; placebo, 29.1 months). The estimated four-year OS rates were 49.6% and 36.3% for durvalumab versus the placebo, while the four-year PFS rates were 35.3% and 19.5%, respectively [34]. These updated exploratory analyses demonstrate durable PFS and sustained OS benefits with durvalumab after chemoradiotherapy.

### 3.6. Cemiplimab

Cemiplimab is an antibody immunotherapy that stimulates an anticancer response via programmed cell death PD-1 blockade. It is the first approved treatment in the US and EU for patients with locally advanced or metastatic cutaneous squamous cell carcinoma who are not candidates for curative surgery or curative radiotherapy [35].

Recently, in the EMPOWER-Lung 1 study, the PD-1 inhibitor cemiplimab was compared with first-line chemotherapy in the treatment of stage IV NSCLC patients. In the population with PD-L1 expression of at least 50%, which consisted of 563 patients, median OS was not reached with cemiplimab (*n* = 283), while it was 14.2 months in the population that received chemotherapy (*n* = 280). The median PFS was 8.2 months with cemiplimab versus 5.7 months with chemotherapy. Significant improvements in OS and PFS were also observed with cemiplimab in the intention-to-treat population, despite a high crossover rate (74%) [36].

The EMPOWER-Lung 1 study implies that cemiplimab monotherapy significantly improved OS and PFS compared to chemotherapy in patients with advanced NSCLC with PD-L1 expression of at least 50%, providing a potential new treatment option for this patient population. Although ICIs present a significant survival benefit for the majority of patients with advanced NSCLC, the ORR is approximately 20%, and the majority of patients do not respond to these therapies, especially monotherapy in NSCLC immunotherapy [37].

We summarized important clinical trials in the field of NSCLC immune monotherapy (Table 1).

## 4. Combination Therapy

Research on immunotherapy for NSCLC has made great progress in the past few years. However, the low ORR of immunotherapy is its shortcoming. The ORR of the first-line monotherapy selection population was 45% (PD-L1 ≥ 50%, approximately 20%), and the ORR of the second-line treatment was only 20%. Moreover, immunotherapy also carries the risk for super-progression (6–29%) and pseudo-progression (4.5%). Therefore, clinical oncologists have always expected to combine conventional treatments with immunotherapy in order to improve efficacy, which we will discuss in this section, and we summarized important clinical trials in the field of combination therapy.

### 4.1. Immunotherapy Combined with Chemotherapy

Immunotherapy research has made great progress in the past few years, and immunotherapy strategies have gradually shifted from second-line monotherapy to first-line and combination therapies, mainly due to the success of multiple studies on combining immunotherapy with chemotherapy.

The breakthrough for immunotherapy combined with chemotherapy in advanced NSCLC first came from the treatment of NS-NSCLC. In a randomized, open-label, phase II cohort of a multicohort study (KEYNOTE-021G), 123 patients were enrolled. Of the 60 patients in the pembrolizumab plus chemotherapy group, 33 (55%) achieved an objective response compared to 18 (29%) of the 63 patients in the chemotherapy alone group [38]. Subsequently, the FDA approved the indication of pembrolizumab combined with chemotherapy for the first-line treatment of advanced non-squamous NSCLC. In 2018, as a confirmatory study of KEYNOTE-021G, the phase III KEYNOTE-189 study with PFS and OS as the primary endpoints once again confirmed the above conclusions [39]. Based on the above research, in August 2018, the FDA approved pembrolizumab in combination with pemetrexed and platinum as first-line treatment of patients with metastatic NS-NSCLC and without EGFR or ALK genomic tumor aberrations. This is the first approved immune combination chemotherapy regimen. The latest KEYNOTE-407 demonstrated that in the first-line treatment of metastatic SCC, pembrolizumab combined with carboplatin and taxane chemotherapy, when compared to chemotherapy alone and regardless of which type of taxane chemotherapy the patient receives, can significantly improve OS, PFS, and ORR, and that it is tolerated by patients [40].

IMpower130 aimed to assess the efficacy and safety of atezolizumab plus carboplatin plus nab-paclitaxel (ACnP) versus chemotherapy (ACnP) alone as a first-line therapy for NS-NSCLC. IMpower130 showed a significant and clinically meaningful improvement in OS and a significant improvement in PFS with atezolizumab plus chemotherapy versus chemotherapy as a first-line treatment for patients with stage IV NS-NSCLC and no ALK or EGFR mutations [41]. This study supports the benefit of atezolizumab, in combination with platinum-based chemotherapy, as a first-line treatment of metastatic NSCLC. Based on this result, ACnP was approved by the EU FDA for the first-line treatment of non-squamous cell carcinoma NSCLC patients without EGFR/ALK changes and was included in the NCCN and ESMO guidelines. The IMpower131 study found that the median PFS of nab-paclitaxel plus carboplatin chemotherapy combined with atezolizumab reached 6.3 months, which was significantly better than that of the chemotherapy group [42]. Subsequently, IMpower132 evaluated the effect of atezolizumab plus carboplatin or cisplatin plus pemetrexed in patients with non-squamous NSCLC. Makoto et al. reported the PFS and ORR of the chemotherapy combined with atezolizumab group as being better than those of the chemotherapy group: The PFS was 7.6 and 5.2 months, respectively [43]. The results of the PFS subgroup analysis showed that most of the subgroups of the combination treatment group showed survival benefits, especially the Asian population, who demonstrated more obvious benefits in terms of PFS.

The application of durvalumab in NSCLC monotherapy was widely investigated in the PACIFIC study. Recently, Sacha et al. studied the effect of durvalumab combined with chemotherapy in NSCLC (SAKK 16/14) [44]; the results showed that the addition of perioperative durvalumab to neoadjuvant chemotherapy in patients with stage IIIA(N2) NSCLC is safe and exceeds the historical data of chemotherapy alone, with a high MPR and an encouraging one-year EFS rate of 73%. Several prospective clinical studies, including this study, have confirmed the good efficacy of neoadjuvant immune combined chemotherapy in patients with stage III NSCLC. However, SAKK 16/14 demonstrated a relatively high rate of adverse events of grade ≥ 3. This may be related to the studies’ neoadjuvant regimen (chemo-sequential PD-L1 monoclonal antibody), which prolonged the neoadjuvant treatment time.

As a new ICI, sintilimab has also received widespread attention in recent years. Yang et al. explored the effect of sintilimab plus pemetrexed and platinum as a first-line treatment for locally advanced or metastatic NS-NSCLC. The results showed that in Chinese patients with previously untreated, locally advanced, or metastatic NS-NSCLC, the addition of sintilimab to chemotherapy with pemetrexed and platinum resulted in a considerably longer PFS than with chemotherapy alone, with manageable safety profiles [45]. This study further expanded the application of ICIs combined with chemotherapy.

With the extensive development of studies on ICIs combined with chemotherapy in NSCLC, people have a deeper understanding of the application of ICIs in NSCLC, and a large number of combined treatment options have been included in the guidelines and expert consensus. In the coming period, a large number of studies of ICIs in combination with chemotherapy will reach their endpoints, and we expect more NSCLC patients to benefit from combined therapy.

### 4.2. Immunotherapy Combined with Radiotherapy

Radiotherapy (RT) is widely used in tumor therapy and is the most effective cytotoxic therapy for solid tumor patients [46]. For patients with locally advanced unresectable disease, RT can be combined with chemotherapy for palliative treatment, which can not only kill tumor cells directly but also release tumor-associated antigen and a series of positive immune stimulation signals in addition to activating cytotoxic T cells to enhance the killing effect of tumor cells [47]. Moreover, radiotherapy can also induce antigenic modulation and immunosuppressive molecule upregulation, weaken the immunogenicity of tumor cells, and promote the inactivation of immune effector cells, finally resulting in the occurrence of immune escape and tolerance [48]. Therefore, it can be speculated that patients with poor clinical radiotherapy may be related to immune escape. As a double-edged sword, the immune activation ability of radiotherapy alone is limited. As previously stated, the mechanism of immunotherapy is to improve the immune microenvironment and to remove the inhibitory effect of the immunosuppressive microenvironment on immune effector cells. Therefore, the effect of radiotherapy plus immunotherapy is worth investigating.

A phase II clinical trial reported that ipilimumab alone or in combination with chemotherapy did not show significant efficacy in patients with chemotherapy-refractory metastatic NSCLC, while ipilimumab in combination with RT increased the immune response [49]. Ipilimumab was associated with an increase in in vitro effects in radiotherapy, suggesting that activation of the immune system led to a non-irradiated response. In another phase III NSCLC study, 709 received the assigned intervention [33]. The results showed that durvalumab had a 24-month OS rate of 66.3%, compared to 55.6%. Durvalumab significantly extended OS compared to the placebo, establishing a standard treatment status of concurrent chemoradiotherapy plus durvalumab adjuvant therapy in locally advanced NSCLC. Willemijn et al. conducted a pooled analysis of two randomized trials—pembrolizumab with or without radiotherapy in metastatic NSCLC—to determine whether radiotherapy improves the immunotherapeutic response in patients with metastatic NSCLC [50]. These promising results indicate that the addition of radiotherapy to pembrolizumab immunotherapy significantly improved treatment efficacy and prognosis for patients with metastatic NSCLC. Recently, Nasser et al., reported a single-center, open-label, randomized, controlled phase II trial, comparing neoadjuvant durvalumab alone with neoadjuvant durvalumab plus stereotactic radiotherapy in patients with early-stage NSCLC [51]. The results show that neoadjuvant durvalumab combined with stereotactic body radiotherapy is well tolerated, safe, and associated with a high major pathological response rate. PD-L1 monoclonal antibody combined with SBRT brings new neoadjuvant treatment options for early resectable NSCLC, especially for stage IIIA patients; its benefits in stage I–II NSCLC may redefine the applicability of neoadjuvant therapy patient. However, it is slightly inferior to the neoadjuvant immune combined chemotherapy regimen. We look forward to the results of subsequent phase III clinical trials as well as to studies exploring the feasibility of immunotherapy combined with radiotherapy strategies in more types of immunological drugs.

In recent years, although radiotherapy combined with immunotherapy has made great progress—it is more effective than radiotherapy alone in clinical practice, although challenges still remain. For example, we also need in-depth research on the contradictory effects caused by radiation therapy to explore the best efficacy and minimal side effects. The toxic effects of combined immunotherapy require further evaluation. For instance, the incidence of radiation or immune-related pneumonia and myocarditis may increase when chest radiotherapy is combined with immunotherapy. Preclinically, excess cardiac mortality has been found in mice treated with cardiac irradiation and PD-1 blockade [52].

Therefore, the radiation method, radiation dose, dose of drug, and other factors should be further considered. Moreover, the best strategy for treatment should be explored. In addition, special considerations are needed for tumor metastasis to certain specific areas, such as the brain, such as addressing whether radiotherapy combined immunotherapy will have stronger toxicity on local tissue through further study. This includes determining whether the specialized tissues or organs could even endure this kind of treatment. Thus, the efficacy and safety of radiotherapy combined with immunotherapy needs to be comprehensively assessed, and more evidence is needed before it can be used in clinical practice.

### 4.3. Dual Immunotherapy

The effectiveness and safety of ICI monotherapy and combination chemotherapy for NSCLC have been confirmed in a large number of trials, and the effectiveness and safety of ICI combination therapy have gradually attracted attention.

It has been found that CTLA-4 and PD-1 ligands block CD3/CD28-mediated glucose metabolism and inhibit the upregulation of Akt activity through different mechanisms [53]. In addition, researchers have also found that anti-PD-1 mainly improves the effector T cell function in the tumor microenvironment, while anti-CTLA-4 mainly inhibits the T cell activation in peripheral lymph nodes and PD-1/PD-L1 and CTLA-4 act on T cells in different stages of cellular activation. Thus, simultaneous targeting of these two immune checkpoints may obtain superpositions or synergies [54]. Preclinical studies have shown that the combination of anti-PD-L1 and anti-CTLA-4 antibodies produces a stronger antitumor effect in mouse models than monotherapy [55].

At present, several studies have been carried out on dual immunotherapy with exciting results [56]. The CheckMate 012 phase I trial randomized 77 patients with advanced NSCLC to receive 3 mg/kg of nivolumab every two weeks in combination with 1 mg/kg of ipilimumab every 12 weeks (*n* = 38) or once every six weeks (*n* = 39) as a first-line treatment. The ORR of the two groups was 47% versus 38%, and the incidence of grade 3–4 adverse reactions was 37% and 33%, respectively. The ORR of patients with PD-L1 ≥ 1% was 57%. These results show that nivolumab plus ipilimumab has a tolerable safety profile as a first-line treatment for advanced NSCLC, with a high response rate and a sustained response that is superior to nivolumab alone. CheckMate 568, an open-label phase II trial, evaluated the efficacy and safety of nivolumab in combination with low-dose ipilimumab as a first-line treatment for advanced/metastatic NSCLC [57]. This trial reconfirmed that nivolumab in combination with low-dose ipilimumab is effective and well tolerated as a first-line treatment for advanced/metastatic NSCLC. Based on this study, Matthew et al. further confirmed that first-line treatment with nivolumab plus ipilimumab resulted in a longer duration of OS than did chemotherapy in NSCLC patients, independent of their PD-L1 expression level (CheckMate 227) [58]. Subsequently, the FDA approved nivolumab (3 mg/kg) combined with ipilimumab (1 mg/kg) for first-line use in advanced NSCLC with PD-L1 ≥ 1%.

Luis et al. further explored the effect of nivolumab plus ipilimumab combined with two cycles of chemotherapy in NSCLC patients (CheckMate 9LA) [59]. The results showed that nivolumab plus ipilimumab with two cycles of chemotherapy provided a significant improvement in OS versus chemotherapy alone and had a favorable risk–benefit profile. These data support use of this regimen as a new first-line treatment option for patients with advanced NSCLC. Based on this study, the FDA approved nivolumab plus ipilimumab combined with two cycles of chemotherapy for first-line use in advanced or relapsed NSCLC [60].

The effect of nivolumab plus ipilimumab in advanced NSCLC has been confirmed, and the latest NEOSTAR study explored the effect of nivolumab plus ipilimumab in the neoadjuvant treatment of operable NSCLC. This phase II randomized study indicates that neoadjuvant nivolumab plus ipilimumab-based therapy enhances pathologic responses, tumor immune infiltrates, and immunologic memory, thus meriting further investigation for used in cases of operable NSCLC [61].

While dual immunotherapy can enhance antitumor effects, it also presents many challenges. There is evidence that blocking the PD-1 pathway alone or in combination with anti-CTLA-4 can produce antitumor effects in NSCLC or SCLC, and combination therapy seems to provide a larger tumor response [62], but treatment-related toxicity is also increased. Therefore, for drug combinations, we should not only focus on its strong efficacy, but also pay attention to the additive effect of drug toxicity. Therefore, the strategy of drug combination needs to be further studied (such as drug dose, individualized drug, and continuous duration), and we need more clinical evidence to guide clinical practice.

### 4.4. Immunotherapy Combined with Targeted Therapy

Targeted therapy can rapidly reduce tumor load, release a large number of tumor antigens, and regulate immunity through a variety of direct or indirect effects [63]. Preclinical evidence shows that the activation of the EGFR signaling pathway may promote tumor cell PD-L1 expression [64]; theoretically, targeted therapy combined with immunotherapy can help enhance antitumor activity. Targeted therapy and immunotherapy are emerging tumor treatment methods, and their combined application in NSCLC has been widely studied.

Zhou et al., explored the effectiveness of different doses of apatinib (250 or 375 mg/day) combined with carrelizumab (200 mg, q2w) for advanced NS-NSCLC patients who have previously received second-line chemotherapy [65]. Compared to carrelizumab or apatinib alone, the combination of the two showed a significant effect in the treatment of advanced NSCLC, indicating that the median PFS of different types of patients can be extended to six to seven months. In terms of safety, the combined program also performed well. Not only did it have fewer adverse events, but it also significantly reduced the reactive capillary hyperplasia (15.6%) caused by carrelizumab. Patients with STK11/KEAP1 mutation might derive more benefits from this combination. This study provides a chemotherapy-free treatment option for patients with advanced lung cancer. The combination of carrelizumab and apatinib is superior to the existing second-line standard chemotherapy in terms of ORR and PFS. Currently, the phase III trial of this study is underway (NCT04203485).

Matthew et al. reported the results from the dose-finding and initial phase II expansion of a phase Ib/II study of lenvatinib plus pembrolizumab in patients with selected advanced NSCLC [66]. The results show that lenvatinib plus pembrolizumab demonstrated a manageable safety profile and promising antitumor activity in NSCLC patients. However, considering the small number of NSCLC patients included in the study, the drawn conclusions need to be verified in further multicenter large-sample clinical trials.

In addition, the role of ICIs in the clinical treatment of EGFR-mutant NSCLC patients remains controversial. A phase III open-label CAURAL trial (NCT02454933) investigated osimertinib plus durvalumab versus osimertinib monotherapy in patients with sensitizing EGFR-TKI and EGFR T790M mutation-positive advanced NSCLC and disease progression after EGFR-TKI therapy [67]. The results of this trial did not show an enhanced effect of targeting EGFR-TKI in combination with ICIs in NSCLC. Instead, grade 3 or higher toxicity occurs.

Moreover, a retrospective analysis—through the FDA adverse event reporting system database—found a higher proportion of interstitial pneumonitis for nivolumab in combination with EGFR-TKI (25.7%) compared to either drug alone, which were less than 5%, with an odds ratio of 5.09 [68].

### 4.5. Combined Anti-Angiogenic Therapy

Tumors are characterized by poor organization, vascular abnormalities, and permeability changes. Angiogenesis is essential for primary tumor growth and has a complex relationship with the immune system [69]. Vascular endothelial growth factor (VEGF) is a key driver of tumor angiogenesis, which is an important process of solid tumor proliferation [70]. Although anti-angiogenic drugs generally do not directly kill tumor cells, they can weaken or prevent the formation of blood vessels in tumors, thereby inhibiting the growth of tumor cells. In fact, studies have found that the VEGF/VEGFR pathway interacts with the immune system [71,72]. By upregulating PD-1 expression in CD8+ T lymphocytes, VEGF binding to VEGFR2 on effector T cells directly inhibits proliferation and cytotoxicity [73]. Therefore, anti-angiogenic therapy combined with ICIs could be promising for the treatment of NSCLC.

Anlotinib is an oral multitarget tyrosine kinase receptor inhibitor (TKI) that selectively inhibits VEGFR 1-3 [74]. Anlotinib has been shown to prolong PFS and OS in patients with refractory advanced NSCLC [75,76]. Anlotinib enhances the role of innate immune cells in the tumor microenvironment, as well as the potential synergistic antitumor efficacy of combined ICIs.

Although the interaction between the tumor immune microenvironment and angiogenesis has been well established, evidence supporting the chemo-free combination of ICIs plus antiangiogenic tyrosine kinase inhibitors in treatment-naive patients with advanced NSCLC is insufficient. Chu et al., reported the efficacy and safety of sintilimab combined with anlotinib as a first-line therapy for advanced NSCLC from a phase 1b trial [77]. A total of 22 patients received sintilimab and anlotinib, and the median follow-up was 15.8 months. Sixteen patients achieved a confirmed partial response with an objective response rate of 72.7% and a disease control rate of 100%. The median PFS was 15 months, and the 12-month PFS rate was 71.4%. This is the first study that assessed an anti-programmed cell death protein 1 antibody combined with a multitarget antiangiogenic tyrosine kinase inhibitor in the first-line setting for NSCLC patients. In view of its encouraging efficacy, durability, and safety profile, sintilimab plus anlotinib represents a novel chemotherapy-free regimen in this patient population.

Bevacizumab is a completely humanized monoclonal antibody that blocksVEGFR1 and VEGFR2 interaction, receiving FDA approval on 26 February 2004, meaning that it is the first approved inhibitor of tumor angiogenesis. Bevacizumab can induce tumor vascular normalization and promote T cell tumor invasion [78,79].

At present, among the relevant studies of bevacizumab in the treatment of NSCLC, the IMpower150 series of studies is one of the most important. In 2018, Socinski et al., demonstrated that atezolizumab plus bevacizumab significantly improved PFS and OS in patients with metastatic NS-NSCLC, regardless of PD-L1 expression and EGFR or ALK gene alteration status [80]. Based on this study, Martin et al. subsequently reported the efficacy of ABCP or atezolizumab plus carboplatin and paclitaxel (ACP) versus BCP in key patient subgroups. The results show that improved survival was achieved in patients treated with ABCP compared to those given BCP in the intention-to-treat population or those with baseline liver metastases [81]. Recently, final OS analyses were presented for EGFR mutations and liver or brain metastases subgroups in the phase III IMpower150 study evaluating ABCP or ACP versus BCP [82]. This final exploratory analysis showed OS benefits for ABCP versus BCP in patients with sensitizing EGFR mutations, including those with prior TKI failures, and with liver metastases, although these results should be interpreted with caution. The impact of ABCP on delaying the development of new brain lesions requires further investigation. According to the IMpower150 study, the FDA approved ABCP for the first-line treatment of non-squamous cell carcinoma patients without EGFR/ALK mutations. In the EU, ABCP was approved for the first-line treatment of non-squamous cancer patients (patients with EGFR/ALK mutations need to undergo targeted therapy), as well as in Japan [83].

In conclusion, the available data provide a strong theoretical basis for the use of combination therapy in treating patients with NSCLC (Table 2). However, there are no additional data for evaluating the safety and efficacy of antiangiogenic agents combined with ICIs in the treatment of NSCLC. Moreover, the therapeutic dose of antiangiogenic therapy in clinical practice varies with tumor type and clinical setting, and further preclinical and clinical studies are needed to optimize antiangiogenic therapy in the age of tumor immunotherapy.

## 5. Biomarkers for Predicting the Efficacy of Immunotherapy

In recent years, immunotherapy based on ICIs has significantly improved the ORR and OS of NSCLC patients and has become an indispensable part of NSCLC treatment. However, the efficacy of monotherapy is limited. Finding specific biomarkers and screening out populations with advantages in immunotherapy before treatment is key to achieving precise treatment. At present, a variety of tissue-based biomarkers have been proven to be effective in predicting the efficacy of NSCLC immunotherapy, mainly including the PD-L1 expression level, tumor mutational burden (TMB), microsatellite instability (MSI), mismatch repair (MMR) gene defects, CD8+ tumor-infiltrating lymphocytes (TILs), T cell inflammatory gene expression profile and driver genes (such as epidermal growth factor receptor (EGFR)), anaplastic lymphoma kinase (ALK), STK11/LKB1 mutation, K-RAS/TP53 co-mutation, murine double minute 2, MDM2/MDM4 amplification, and EGFR amplification [84]. Among them, the high-level evidence is supported by the expression level of PD-L1 and the TMB in tumor tissues. In the next section, we summarized important biomarkers in immunotherapy.

### 5.1. PD-L1

PD-L1 expression is the preferred biomarker for predicting the efficacy of ICIs. Because the high positive rate of PD-L1 is often accompanied by high levels of T lymphocyte infiltration [85], detection of the PD-L1 expression level in tumor tissues is the most direct method for predicting the patient response to PD-1/PD-L1 inhibitors. A systematic review collected 19 studies that evaluated the effect of PD-L1 expression in patients on the effectiveness of ICIs during the treatment of advanced NSCLC. Most of the evidence shows that compared to patients with low PD-L1 expression, patients with high expression who received anti-PD-1/PD-L1 drugs (nivolumab, pembrolizumab, duvalizumab, atilizumab, and avelumab) benefitted more when they were treated as single agents [86]. The PD-L1 expression level is relatively mature in predicting the efficacy of ICIs, and it has become an important basis for clinicians to formulate immunotherapy programs for NSCLC patients.

Notably, a proportion of patients with negative PD-L1 expression can also benefit from PD-1/PD-L1 inhibitor therapy [87]. As mentioned before, PD-L1 has biological heterogeneity; it can be expressed not only by tumor cells, but also by immune cells and some inflammatory cells. Studies have shown that PD-L1 expression also differs between inter- and intratumoral tissues and even changes with treatment [88]. Similarly, it has been reported that the expression of PD-L1 may be inconsistent in sections of the same tissue, and the expression of PD-L1 varies up to 4 times in different regions of the same tissue [89].

Furthermore, the corresponding threshold of PD-L1 in different tumors has been reported differently [90]. CheckMate 017 reported that PD-L1 expression was not associated with the efficacy of anti-PD-1/PD-L1 inhibitors in lung squamous cell carcinoma. In the OAK study, the improvement in the survival of the atezolizumab immunotherapy group was superior to that in the docetaxel chemotherapy group regardless of the PD-L1 expression level [29]. This result reflects that the correlation between PD-L1 expression and therapeutic effect is not completely consistent. A recent study linked TPS for PD-L1 expression with rates of disease control (DCR, partial response, and disease stability) and PFS after treatment with nivolumab or pembrolizumab. The results show that high PD-L1 expression was associated with significantly increased DCR and prolonged PFS in NSCLC patients treated with nivolumab or pembrolizumab. Although the sample size was small in this small retrospective study, the results once again confirm the potential of PD-L1 as a predictive biomarker [91].

Although it is controversial that PD-L1 is the best predictor of immunotherapy efficacy due to the occurrence of some special conditions, PD-L1 detection is still of great guiding significance for patients undergoing immunotherapy. On the one hand, clinicians must understand the guiding significance of the PD-L1 test in the treatment of NSCLC, be familiar with the difference between concomitant and complementary diagnosis and be able to skillfully interpret PD-L1 test reports. On the other hand, for pathologists, the quality and source of PD-L1 tissue specimens must be strictly monitored during PD-L1 detection, and a more appropriate and systematic diagnosis and reporting process should be established. This system can not only be used for extensive PD-L1 detection but can also be combined with other biological detection materials for comprehensive analysis. In addition, PD-L1 expression is regulated by multiple mechanisms [92], including the MAPK and PI3K or Akt pathways, transcription factors HIF1, STAT3, and NFkB, and epigenetic factors [93]. Meanwhile, the changes in PD-L1 before and after treatment should be considered in clinical application. Whether patients have positive or negative PD-L1 expression should be taken into consideration because the immunohistochemical analysis of PD-L1 is also different, which also affects the detection of PD-L1 expression. Therefore, the detection method of PD-L1 also needs to be optimized and standardized.

### 5.2. TMB

Tumor mutation burden (TMB) refers to the relative number of mutations in given tumor tissue; if more nonsynonymous mutations are present in the tumor, more neoantigens emerge, and the PD-1/PD-L1 axis becomes involved in blocking the immune response, thus affecting the response of tumor cells to ICIs.

Rizvi et al. found that an elevated nonsynonymous mutation burden, including DNA repair mutations, a molecular smoking signature, and a high neoantigen load, was significantly associated with the clinical activity of pembrolizumab in NSCLC patients. The proportion of mutations in NSCLC patients who responded to pembrolizumab treatment was much higher than in patients who did not respond to pembrolizumab treatment. Moreover, in the same study, it was concluded that the genomic landscape of lung cancers shapes the response to anti-PD-1 therapy [94]. In 2017, a retrospective study assessed the objective response rate between the TMB and PD-1 inhibition. The results showed that the ORR was positively correlated with the TMB levels in 27 types of tumors treated with PD-1 or PD-L1 inhibitors [95].

Since then, the results of the CheckMate-227 clinical trial have shown that regardless of the PD-L1 expression status of NSCLC patients with a high TMB (≥10 mutations/Mb), the combination of nivolumab and ipilimumab as a first-line treatment significantly prolongs the patient’s PFS [96]. Based on the results of this study, the NCCN-NSCLC expert group listed the TMB as a biomarker in the first edition of the 2019 NCCN guidelines, which is believed to help screen patients with metastatic NSCLC suitable for the first-line treatment of nivolumab with ipilimumab, but also stated that there is currently no standard TMB measurement method [97]. More importantly, many studies have demonstrated that PD-L1 and the TMB are biomarkers associated with the tumor response to combined ICIs [23,94,95,98]. Therefore, the combination of PD-L1 expression and the TMB is considered a promising biomarker for assessing patient survival and response to precise immunotherapy.

Taken together, many studies have suggested that the TMB is a reliable biomarker for immunotherapy; however, clinical challenges are inevitable. Currently, the method used to detect the TMB is genome analysis, including whole-genome sequencing, whole-exon sequencing, and selective gene sequencing. The biggest disadvantage of the TMB precluding its use as an excellent marker of the immunotherapy response rate is that it is difficult to be quantified and standardized. Moreover, gene mutations are different in different tumors, and the critical value of the TMB in different tumors is not clear. Therefore, it is necessary to explore and determine a relatively reliable quantification method and standard for the TMB in different tumors to test its predictive significance in immunotherapy.

### 5.3. TILs

Tumor-infiltrating lymphocytes (TILs) comprise one of the components of the tumor microenvironment—specifically, the heterogeneous group of immune inflammatory cells expressing various activated antigens [99]. The importance of TILs is gaining increasing recognition, especially for their predictive role in cancer treatment. Cancer cells can inactivate TILs in a variety of ways to evade immune surveillance and further develop tumor tissues [100,101]. Evidently, this phenomenon adversely affects the effectiveness of antitumor therapy.

At present, there is considerable evidence that TILs can predict the treatment and prognosis of tumors. For example, CD8+ T cell tumor-infiltrating lymphocytes show satisfactory predictive efficacy in immunotherapy [102]. Evidence shows that the concentration of CD8+ and FOXP3+ Treg TIL in the tumor background can be regarded as predictive markers of the platinum-based neoadjuvant chemotherapy response in patients with advanced NSCLC [103,104]. Chen et al. studied the TILs in the baseline tumor tissues of 25 patients with early stage and 35 patients with advanced NSCLC and discovered a new subgroup accumulating in the TME, namely, CD8+ TILs. They believed that CD8+ TILs in the baseline tumor tissues of NSCLC patients and the corresponding abundance of ICIs is related to resistance to ICI treatment [105]. This study suggested a strategy to overcome resistance to ICIs: the abundance of CD8+ TILs in the baseline tumor tissues of NSCLC patients may be used as a potential marker to predict the efficacy of ICIs. Markers for NSCLC patients treated with ICIs. The 2020 AACR annual meeting announced the research data of a phase I clinical trial based on TILs for the treatment of advanced NSCLC. The study included 12 patients with advanced NSCLC who received nivolumab treatment and whose disease progressed. The researchers extracted the tumor lesions from these patients, in which TILs were amplified in vitro and then infused back into the patient. After receiving the treatment, most patients underwent an average reduction of 38% in the diameter of the tumor upon the first reexamination of the chest CT [106]. The results of this study imply that TILs not only provide a promising treatment model for NSCLC patients with resistance after ICI treatment but also confirm the value of TILs as predictive biomarkers. The above results show that TILs, as a new marker for predicting the efficacy of ICIs, may be of great significance for guiding immunotherapy.

Although TILs have been poorly studied as a predictive marker in NSCLC, the available evidence suggests that they are a promising biomarker. The current TIL subpopulation techniques mainly include immunohistochemistry or flow cytometry, but these two techniques have limitations. Therefore, the next challenge to overcome is to develop standardized, repeatable, and effective methods, as TIL scoring in large-scale clinical trials and routine histopathological practice is of great significance in predicting prognosis and treatment response.

### 5.4. Driver Gene Mutations

At present, it is known that NSCLC patients with positive driver genes have a very low response to immunotherapy, and some driver gene mutations have been the key exclusion criteria for the clinical application of ICIs. Carrying EGFR mutations or ALK rearrangement is significantly correlated with the low response of NSCLC patients to ICIs [107,108,109]. In the 2020 NCCN guidelines, the NSCLC expert group proposed that NSCLC patients with PD-1 expression levels ≥1% but with driver gene mutations should first choose targeted therapies instead of ICIs. Before using PD-1 or PD-L1 inhibitors, the status of EGFR and ALK should at least be determined. It would be more ideal if it can be supplemented with negative testing of “ROS1 fusion” and “BRAF mutation” as a criterion [97]. Other mutated genes that are not clearly indicated in the guidelines may also be related to the response level of ICIs. Some rare tumor gene mutations may also predict the response level of NSCLC patients to ICIs. Among NSCLC patients, phosphatidylinositol-4,5-bisphosphate 3-kinase catalytic subunit alpha (PIK3CA), EGFR, or STK11 mutations do not respond to ICIs, while KRAS, tumor protein P53 (TP53) mutants, and mesenchymal-to-epithelial transition factor (MET) gene exon 14 skipping mutations respond well to ICIs [110]. In the future, more research is needed to explore the potential biomarkers of many tumor driver genes that can be used to predict the efficacy of ICIs.

### 5.5. Other Biomarkers

Peripheral blood analysis is a non-invasive method with rich components and important clinical values, such as a variety of inflammatory indicators. In addition, this method has good potential to predict treatment outcomes after immunotherapy. Peripheral blood lymphocyte absolute count (ALC) has good predictive significance [111]. The applicability of LDH as a predictive biomarker has also been confirmed. Despite limited evidence, the results show that patients with elevated LDH levels also responded to ICIs [112]. In addition, the predictive value of microRNA, neoantigens, MSI, extracellular vesicles, gut metabolism, etc., for the efficacy of ICIs has also been widely studied, and some biomarkers perform well in predicting efficacy when used in combination [113].

To conclude, actively looking for predictive biomarkers in immunotherapy is the purpose of the screening of beneficiaries, which is not only the connotation of the targeted biological model in addition to promoting the development of an accurate medical model. Due to the immune system being too complex to be explained by a single biomarker, researchers should not only focus on a single prediction significance of biomarkers—integrated thinking is also very critical. Therefore, the value of the combined prediction of biomolecules and specific cell populations is worth pondering, such as inflammatory factors in the TME, Tregs, and some special genes. More importantly, practice is the only way to test whether this hypothesis is correct. Thus, whether the combined use of biomarkers is better than single biomarkers also needs to be considered and confirmed. Finally, we have to look at the clinical efficacy of biomarkers, as well as the practical difficulty and associated cost. In recent years, the American Society of Clinical Oncology has increasingly focused on the value of cancer treatments, mentioning their benefit/cost ratio [114], because the ultimate goal is to provide a higher level of care for patients and determine what is best for the majority of patients (Table 3).

## 6. Challenges and Prospects of NSCLC Immunotherapy

Although breakthroughs have been made in the treatment of tumors such as NSCLC, checkpoint suppression-mediated immunotherapy still has shortcomings and deficiencies. On the one hand, the beneficiaries are still in the minority—only 20–30% of cancer patients respond to ICIs treatment [117]. On the other hand, toxicity superposition, economic burden, special crowd of NSCLC, and drug resistance have always existed.

### 6.1. Drug Toxicity

ICIs are a double-edged sword. While killing tumors, ICIs can also cause a series of immune-related adverse events (irAEs). Multiple studies have shown that irAEs associated with anti-PD-1 and anti-PD-L1 may include fatigue, rashes, diarrhea, pruritus, decreased appetite, nausea, colitis, hepatitis, thyroiditis, neuropathy, nephritis, myocarditis, and inflammatory arthritis [118,119,120]. CheckMate 568, a study about first-line nivolumab plus ipilimumab treatment in advanced NSCLC, showed that 29% of patients had grade 3 or 4 treatment-related adverse events, and the causes of treatment-related death included immune-mediated hepatitis associated toxicity with the progression of tumor liver metastasis, grade 4 bilirubin elevation, grade 1 aspartate aminotransferase elevation, and grade 2 alanine aminotransferase elevation [57]. In addition, checkpoint inhibitor pneumonia (CIP) is a growing concern. The clinical presentation of CIP patients is nonspecific and is characterized by dyspnea, cough, fever, chest pain, and a gradual decrease in exercise tolerance [121]. The previous clinical incidence of CIP in trials has been reported to be 3–5% [122,123]. Subsequently, the results of another study showed a 19% incidence of CIP. The data also suggested that tumor histological type and ICIs therapy may be risk factors associated with CIP [124]. Corticosteroids have been shown to improve symptoms in CIP patients [119,125].

Steroid hormones can treat most irAEs, especially mild irAEs, but there are indeed some refractory irAEs that are resistant to steroids or that cannot be dealt with by steroids alone. The application of other immunosuppressive agents, especially specific immunosuppression, is needed.

### 6.2. Drug Resistance

The drug resistance of ICIs is one of the great challenges facing tumor immunotherapy at present. Tumor immunotherapy resistance is the result of the interaction of host, tumor cells and the immune microenvironment. Treg cells, myeloid-derived suppressor cells (MDSCs), M2 macrophages, and other inhibitory factors in the tumor microenvironment can also lead to immunotherapy resistance. Treg cells can secrete inhibitory cytokines, including IL-10, IL-35, and TGF-β. Animal experiments have confirmed that the elimination of Treg cells in the tumor microenvironment can enhance antitumor immunity [126]. The associated filtration of hypoxia and immunosuppressive cells in tumors appears to be the main cause of angiogenesis recurrence and drug resistance [127].

At present, a combination therapy strategy is the most important and effective measure to delay or reverse immune resistance. Various combination therapies, such as the combination of other types of immunotherapy drugs, chemotherapy, anti-angiogenic therapy drugs, and radiotherapy, can also eliminate the cause of drug resistance through the combined regulation of intestinal flora, thus improving the efficacy of ICIs. However, other problems arise under various combinations of therapies, namely, patient tolerance and financial burden.

With the continuous promotion and application of immunotherapy, single-drug resistance has become an unavoidable problem. The increasingly in-depth research on the mechanism of immune resistance also provides new ideas for the selection of immunotherapy populations and strategies for reversing immune resistance. Targeting strategies based on the type of drug resistance of patients can improve treatment efficiency, reduce treatment costs, and bring about long-term practical benefits to cancer patients. At the same time, it is undeniable that there are still deficiencies in the research of immunological resistance. How to predict immunological resistance and the timing of restarting immunotherapy have not been properly answered, and further exploration is needed.

All of above indicate that doctors should not only be concerned about the strong antitumor effect of combined immunotherapy but should also pay attention to the superposition of drug toxicity, drug resistance, and special populations. There is evidence that blocking the PD-1 pathway alone or in combination with anti-CTLA-4 monoclonal antibody can produce antitumor effects in either NSCLC or SCLC, and combination therapy provides a larger tumor response; however, therapy-related toxicity also increases [62]. Therefore, challenges associated with immunotherapy such as toxicity, including in combination with other drugs, need to be addressed urgently. To explore the optimal drug dose, continuous effective time, individual drug use, drug resistance mechanism, and other aspects are the directions of future research. We need more clinical evidence to guide clinical practice.

### 6.3. Special Crowd of NSCLC

In general, ICI treatment in most patients is well tolerated, and even some adverse events can be resolved with timely management. However, there is a lack of reliable evidence for specific populations, especially patients with pre-existing autoimmune diseases, organ transplants, or chronic viral infections. No age-appropriate safety data have been published for immunotherapy or combination immunotherapy, hindering the wider clinical application of ICIs. ICIs may increase the risk of irAEs in the elderly due to impaired organ function and immune system function, and earlier immunotherapy is not better for this group of people [128]. Therefore, the safety of immunotherapy needs to be demonstrated through more supporting data, and the indications and contraindications for each drug and treatment strategy need to be explained in detail. Some subgroups, such as patients with negative PD-L1 expression and low TMB, benefit little from immunotherapy combinations based on current clinical evidence. For them, standard platinum chemotherapy may still be the treatment of choice.

In the age of immunotherapy, most clinical studies have not paid attention to the immunotherapy options of special populations such as elderly patients, smoking status, hormone use, liver metastasis, and brain metastasis, and even those who are excluded due to having sensitive mutations in driver genes and/or special diseases. However, in the real world, these special populations are not high in number, and population characteristics are not only a supplement to biomarkers but also help to further accurately select the dominant populations for immunotherapy. We believe that it is necessary to strengthen the research on the efficacy and side effects of immunotherapy for special populations.

## 7. Conclusions

Numerous clinical and experimental studies have found the efficacy and safety of immunotherapy to be positive. The immunotherapy of NSCLC is undergoing an important shift from the traditional immune enhancement method based on activating the systemic immune response to a tumor-induced immune escape mechanism and a more effective and less toxic immune normal against the tumor microenvironment. Great progress has been made in the research of immune monotherapy and combination therapy. Combined therapy plays a particularly important role in improving the effectiveness of immunotherapy, expanding the beneficiary population, and overcoming drug resistance. The era of NSCLC immunotherapy seems to have come, but the choice of combination therapy, combination mechanism, and choice of biomarkers need to be further explored. Immunotherapy still has problems such as limited application range, non-standard measurement methods, and reliability to be solved. In addition, the high cost of ICI is also a current problem. We believe that future NSCLC immunotherapy should not only aim at improving anti-tumor immunity, but also understand the specific deficiencies of tumor immunity, and then standardize them to selectively modify specific types of tumor immunity and carry out anti-tumor immunity in the right place, rather than exacerbating systemic immune responses, thus increasing the risk of irAE. In the future, biomarkers for predicting the efficacy of ICI should be determined through whole-genome sequencing and epigenetic analysis, and a comprehensive prediction model of multiple biomarkers and multiple monitoring technologies should be constructed to comprehensively evaluate the patient’s tumor immune status and formulate a personalized precise combined treatment strategy. In addition, we should continue to explore the drug resistance mechanism and new targets of ICIs and develop new drugs to achieve precise immunotherapy for NSCLC patients.

## Figures and Tables

**Table 1:** Current immune monotherapy trials in NSCLC.

Drugs	Target	Trials	Pathological Type	Details	Endpoint	Ref.
Nivolumab	PD-1	CheckMate 057	NS-NSCLC	Nivolumab vs. Docetaxel	Median OS: 12.2 months vs. 9.4 months	[21]
		CheckMate 017	Squamous NSCLC	Nivolumab vs. Docetaxel	Median OS: 9.2 months vs. 6.0 months	[22]
Pembrolizumab	PD-1	KEYNOTE-001	NSCLC	Treatment-naive vs. Previously treated	Median OS: 22.3 months vs. 10.5 months	[25]
		KEYNOTE-024	NSCLC	Pembrolizumab vs. Platinum-based chemotherapy	Median OS: 26.3 months vs. 13.4 months	[26]
Atezolizumab	PD-L1	POPLAR	NSCLC	Atezolizumab vs. Docetaxel	Median OS: 12.6 months vs. 9.7 months	[28]
		OAK	NSCLC	Atezolizumab vs. Docetaxel	Median OS: 13.3 months vs. 9.8 months	[29]
		IMpower110	NSCLC with PD-L1- positive	Atezolizumab vs. Platinum-based chemotherapy	Median OS: 20.2 months vs. 13.1 months	[30]
Avelumab	PD-L1	JAVELIN Lung 100	NSCLC	Avelumab	Median OS: 14.1 months	[31]
		JAVELIN Lung 200	NSCLC	Avelumab vs. Docetaxel	Two-year OS rates: 29.9% vs. 20.5%	[32]
Durvalumab	PD-L1	PACIFIC	NSCLC	Durvalumab vs. Placebo	Two-year OS rates: 66.3% vs. 55.6%	[33]
			NSCLC	Durvalumab vs. Placebo	Four-year OS rates: 49.6% vs. 36.3%	[34]
Cemiplimab	PD-1	EMPOWER-Lung 1	NSCLC with PD-L1 > 50%	Cemiplimab vs. Platinum-based chemotherapy	Median PFS: 8.2 months vs 5.7 months	[36]

Abbreviations: NSCLC, non-small cell lung cancer; NS-NSCLC, non-squamous non-small cell lung cancer; PD-1, programmed cell death receptor-1; PD-L1, programmed cell death ligand 1; OS, overall survival; PFS, progression-free survival.

**Table 2:** Current immune combination therapy trials in NSCLC.

Drugs	Trials	Pathological Type	Details	Endpoint	Ref.
Immunotherapy + Chemotherapy	KEYNOTE-021G	NSCLC	Pembrolizumab + Platinum-doublet chemotherapy vs. Platinum-doublet chemotherapy	ORR: 55% vs. 29%	[38]
	KEYNOTE-189	NS-NSCLC	Pembrolizumab + Pemetrexed-platinum vs. Placebo + Pemetrexed-platinum	Two-year Median OS: 22.0 months vs. 10.7 months	[39]
	KEYNOTE-407	Squamous NSCLC	Pembrolizumab + Carboplatin and paclitaxel vs Placebo + Carboplatin and paclitaxel	Two-year Median OS: 17.1 months vs. 11.6 months	[40]
	IMpower130	NS-NSCLC	Atezolizumab + Carboplatin plus nab-paclitaxel vs Carboplatin plus nab-paclitaxel	Median OS: 18.6 months vs. 13.9 months	[41]
	IMpower131	Squamous NSCLC	Atezolizumab + Carboplatin plus nab-paclitaxel vs. Carboplatin plus nab-paclitaxel	Median PFS: 6.3 months vs. 5.6 months	[42]
	IMpower132	NS-NSCLC	Atezolizumab + Pemetrexed vs. Pemetrexed	Median PFS: 7.6 months vs. 5.2 months	[43]
	SAKK 16/14	NSCLC	Durvalumab plus Cisplatin and docetaxel	One-year EFS rate: 73%	[44]
	NCT03607539	NS-NSCLC	Sintilimab plus Pemetrexed and platinum vs. Placebo plus Pemetrexed and platinum	Median PFS: 8.9 months vs. 5.0 months	[45]
Immunotherapy + Radiotherapy	NCT02221739	NSCLC	Ipilimumab and Radiotherapy	ORR: 18%	[49]
	NCT02125461	NSCLC	Durvalumab + Chemoradiotherapy vs. Chemoradiotherapy	Two-year OS rates: 66.3% vs. 55.6%	[33]
	NCT02492568 and NCT02444741	NSCLC	Pembrolizumab + Radiotherapy vs. Pembrolizumab	Two-year Median OS: 19.2 months vs. 8.7 months	[50]
Dual Immunotherapy	CheckMate 012	NSCLC	Nivolumab (every 2 week) + Ipilimumab (every 12 week) vs. Nivolumab (every 2 week) + Ipilimumab (every 6 week)	ORR: 47% vs. 38%	[56]
	CheckMate 568	NSCLC	Nivolumab + Ipilimumab	ORR: 30%	[57]
	NCT02477826	NSCLC	Nivolumab + Ipilimumab vs. Platinum doublet chemotherapy	Median OS:17.1 months vs. 13.9 months	[58]
	CheckMate 9LA	NSCLC	Nivolumab + Ipilimumab vs. Platinum doublet chemotherapy	Median OS:14.1 months vs. 10.7 months	[59]
Immunotherapy + Targeted therapy	NCT04203485	NS-NSCLC	Camrelizumab + Apatinib	ORR: 30.9%	[65]
	NCT02501096	NSCLC	Pembrolizumab + Lenvatinib	ORR: 33.0%	[66]
Immunotherapy + Anti-Angiogenic Therapy	NCT03628521	NSCLC	Sintilimab + Anlotinib	ORR: 72.7%	[77]
	IMpower150	NS-NSCLC	ABCP vs BCP	Median OS: 19.2 months vs. 14.7 months	[80]

Abbreviations: NSCLC, non-small cell lung cancer; NS-NSCLC, non-squamous non-small cell lung cancer; OS, overall survival; PFS, progression-free survival; EFS, event-free survival; ORR, objective response rate; BCP, bevacizumab plus carboplatin plus paclitaxel; ABCP, atezolizumab plus bevacizumab plus carboplatin plus paclitaxel.

**Table 3:** NSCLC immunotherapy efficacy prediction biomarkers.

Biomarkers	Details	Ref.
PD-L1 expression	NCCN-NSCLC guidelines have been recommended for PD-L1 testing from 2A to Category 1 since 2019. Using immunohistochemical methods, PD-L1 has ≥1% tumors in 60% of advanced NSCLC Cell expression, high level expression in 25–30% of patients (≥50% tumor cells).	[115,116]
TMB	NCCN-NSCLC expert group listed the TMB as a biomarker in the first edition of the 2019 NCCN guidelines. In 2020, TMB was approved by the FDA as a biomarker for pan-solid tumor immunotherapy.	[96]
TILs	TILs provide a promising treatment model for NSCLC patients with resistance after ICI treatment and confirm the value of TILs as predictive biomarkers, but its application value needs further exploration.	[104,105,106]
Driver gene mutations	Carrying EGFR mutations or ALK rearrangement is significantly correlated with the low response of NSCLC patients to ICIs. PIK3CA, EGFR, or STK11 mutations do not respond to ICIs, KRAS, TP53 mutants, and MET gene exon 14 skipping mutations respond well to ICIs.	[107,108,109,110]
Other biomarkers	The predictive value of microRNA, neoantigens, MSI, extracellular vesicles, gut metabolism, etc., for the efficacy of ICIs has also been widely studied, and some biomarkers perform well in predicting efficacy when used in combination.	[111,112,113]

Abbreviations: NSCLC, non-small cell lung cancer; NCCN, National Comprehensive Cancer Network; PD-1, programmed cell death receptor-1; PD-L1, programmed cell death ligand 1; TMB, tumor mutation burden; FDA, Food and Drug Administration; TILs, tumor-infiltrating lymphocytes; ICI, immune checkpoint inhibitor; EGFR, epidermal growth factor receptor; ALK, anaplastic lymphoma kinase; PIK3CA, phosphatidylinositol-4,5-bisphosphate 3-kinase catalytic subunit alpha; MET, mesenchymal-to-epithelial transition factor; MSI, microsatellite instability.

## Data Availability

Not applicable.

## References

[1] Bray F., Ferlay J., Soerjomataram I., Siegel R.L., Torre L.A., Jemal A. (2018). Global cancer statistics 2018: GLOBOCAN estimates of incidence and mortality worldwide for 36 cancers in 185 countries. CA Cancer J. Clin..

[2] Beasley M.B., Brambilla E., Travis W.D. (2005). The 2004 World Health Organization classification of lung tumors. Semin. Roentgenol..

[3] Sands J., Tammemägi M.C., Couraud S., Baldwin D.R., Borondy-Kitts A., Yankelevitz D., Lewis J., Grannis F., Kauczor H.-U., von Stackelberg O. (2021). Lung Screening Benefits and Challenges: A Review of The Data and Outline for Implementation. J. Thorac. Oncol..

[4] Passaro A., Mok T., Peters S., Popat S., Ahn M.-J., de Marinis F. (2021). Recent Advances on the Role of EGFR Tyrosine Kinase Inhibitors in the Management of NSCLC With Uncommon, Non-Exon 20 Insertions, EGFR Mutations. J. Thorac. Oncol..

[5] A Thai A., Solomon B.J., Sequist L.V., Gainor J.F., Heist R.S. (2021). Lung cancer. Lancet.

[6] Behrend S.J., Giotopoulou G.A., Spella M., Stathopoulos G.T. (2021). A role for club cells in smoking-associated lung adenocarcinoma. Eur. Respir. Rev..

[7] Tang A., Ahmad U., Toth A.J., Bourdakos N., Raja S., Raymond D.P., Blackstone E.H., Murthy S.C. (2021). Non-small cell lung cancer in never-and ever-smokers: Is it the same disease?. J. Thorac. Cardiovasc. Surg..

[8] Steven A., Fisher S.A., Robinson B.W., Fong K.M., Van Zandwijk N. (2016). Immunotherapy for lung cancer. Respirology.

[9] Van Coillie S., Wiernicki B., Xu J. (2020). Molecular and Cellular Functions of CTLA-4. Adv. Exp. Med. Biol..

[10] Garcia-Diaz A., Shin D.S., Moreno B.H., Saco J., Escuin-Ordinas H., Rodriguez G.A., Zaretsky J.M., Sun L., Hugo W., Wang X. (2017). Interferon Receptor Signaling Pathways Regulating PD-L1 and PD-L2 Expression. Cell Rep..

[11] Dong H., Strome S.E., Salomao D.R., Tamura H., Hirano F., Flies D.B., Roche P.C., Lu J., Zhu G., Tamada K. (2002). Tumor-associated B7-H1 promotes T-cell apoptosis: A potential mechanism of immune evasion. Nat. Med..

[12] Dong H., Zhu G., Tamada K., Chen L. (1999). B7-H1, a third member of the B7 family, co-stimulates T-cell proliferation and interleukin-10 secretion. Nat. Med..

[13] Chen D., Irving B.A., Hodi F.S. (2012). Molecular pathways: Next-generation immunotherapy--inhibiting programmed death-ligand 1 and programmed death-1. Clin. Cancer Res..

[14] Sun C., Mezzadra R., Schumacher T.N. (2018). Regulation and Function of the PD-L1 Checkpoint. Immunity.

[15] Qu J., Mei Q., Liu L., Cheng T., Wang P., Chen L., Zhou J. (2021). The progress and challenge of anti-PD-1/PD-L1 immunotherapy in treating non-small cell lung cancer. Ther. Adv. Med. Oncol..

[16] Lamberti G., Andrini E., Sisi M., Rizzo A., Parisi C., Di Federico A., Gelsomino F., Ardizzoni A. (2020). Beyond EGFR, ALK and ROS1: Current evidence and future perspectives on newly targetable oncogenic drivers in lung adenocarcinoma. Crit. Rev. Oncol. Hematol..

[17] Xuan Y., Guan M., Zhang S. (2021). Tumor immunotherapy and multi-mode therapies mediated by medical imaging of nanoprobes. Theranostics.

[18] Ellis P.M., Vella E.T., Ung Y.C. (2017). Immune Checkpoint Inhibitors for Patients with Advanced Non-Small-Cell Lung Cancer: A Systematic Review. Clin. Lung Cancer.

[19] Khanna P., Blais N., Gaudreau P.-O., Corrales-Rodriguez L. (2017). Immunotherapy Comes of Age in Lung Cancer. Clin. Lung Cancer.

[20] Deeks E.D. (2014). Nivolumab: A review of its use in patients with malignant melanoma. Drugs.

[21] Borghaei H., Paz-Ares L., Horn L., Spigel D.R., Steins M., Ready N.E., Chow L.Q., Vokes E.E., Felip E., Holgado E. (2015). Nivolumab versus Docetaxel in Advanced Nonsquamous Non-Small-Cell Lung Cancer. N. Engl. J. Med..

[22] Brahmer J., Reckamp K.L., Baas P., Crinò L., Eberhardt W.E.E., Poddubskaya E., Antonia S., Pluzanski A., Vokes E.E., Holgado E. (2015). Nivolumab versus Docetaxel in Advanced Squamous-Cell Non-Small-Cell Lung Cancer. N. Engl. J. Med..

[23] Carbone D.P., Reck M., Paz-Ares L., Creelan B., Horn L., Steins M., Felip E., Heuvel M.M.v.d., Ciuleanu T.-E., Badin F. (2017). First-Line Nivolumab in Stage IV or Recurrent Non-Small-Cell Lung Cancer. N. Engl. J. Med..

[24] Borghaei H., Gettinger S., Vokes E.E., Chow L.Q.M., Burgio M.A., Carpeno J.d.C., Pluzanski A., Arrieta O., Frontera O.A., Chiari R. (2021). Five-Year Outcomes from the Randomized, Phase III Trials CheckMate 017 and 057: Nivolumab Versus Docetaxel in Previously Treated Non-Small-Cell Lung Cancer. J. Clin. Oncol..

[25] Garon E.B., Hellmann M.D., Rizvi N.A., Carcereny E., Leighl N.B., Ahn M.-J., Eder J.P., Balmanoukian A.S., Aggarwal C., Horn L. (2019). Five-Year OS for Patients with Advanced Non-Small-Cell Lung Cancer Treated with Pembrolizumab: Results from the Phase I KEYNOTE-001 Study. J. Clin. Oncol..

[26] Reck M., Rodríguez-Abreu D., Robinson A.G., Hui R., Csőszi T., Fülöp A., Gottfried M., Peled N., Tafreshi A., Cuffe S. (2021). Five-Year Outcomes with Pembrolizumab Versus Chemotherapy for Metastatic Non-Small-Cell Lung Cancer With PD-L1 Tumor Proportion Score ≥ 50. J. Clin. Oncol..

[27] Mok T.S.K., Wu Y.-L., Kudaba I., Kowalski D.M., Cho B.C., Turna H.Z., Castro G., Srimuninnimit V., Laktionov K.K., Bondarenko I. (2019). Pembrolizumab versus chemotherapy for previously untreated, PD-L1-expressing, locally advanced or metastatic non-small-cell lung cancer (KEYNOTE-042): A randomised, open-label, controlled, phase 3 trial. Lancet.

[28] Fehrenbacher L., Spira A., Ballinger M., Kowanetz M., Vansteenkiste J., Mazieres J., Park K., Smith D., Artal-Cortes A., Lewanski C. (2016). Atezolizumab versus docetaxel for patients with previously treated non-small-cell lung cancer (POPLAR): A multicentre, open-label, phase 2 randomised controlled trial. Lancet.

[29] Rittmeyer A., Barlesi F., Waterkamp D., Park K., Ciardiello F., von Pawel J., Gadgeel S.M., Hida T., Kowalski D.M., Dols M.C. (2017). Atezolizumab versus docetaxel in patients with previously treated non-small-cell lung cancer (OAK): A phase 3, open-label, multicentre randomised controlled trial. Lancet.

[30] Herbst R.S., Giaccone G., de Marinis F., Reinmuth N., Vergnenegre A., Barrios C.H., Morise M., Felip E., Andric Z., Geater S. (2020). Atezolizumab for First-Line Treatment of PD-L1-Selected Patients with NSCLC. N. Engl. J. Med..

[31] Verschraegen C.F., Jerusalem G., McClay E.F., Iannotti N., Redfern C.H., Bennouna J., Chen F.L., Kelly K., Mehnert J., Morris J.C. (2020). Efficacy and safety of first-line avelumab in patients with advanced non-small cell lung cancer: Results from a phase Ib cohort of the JAVELIN Solid Tumor study. J. Immunother. Cancer..

[32] Park K., Özgüroğlu M., Vansteenkiste J., Spigel D., Yang J.C.-H., Ishii H., Garassino M., de Marinis F., Szczesna A., Polychronis A. (2021). Avelumab Versus Docetaxel in Patients with Platinum-Treated Advanced NSCLC: 2-Year Follow-Up from the JAVELIN Lung 200 Phase 3 Trial. J. Thorac. Oncol..

[33] Antonia S.J., Villegas A., Daniel D., Vicente D., Murakami S., Hui R., Kurata T., Chiappori A., Lee K.H., de Wit M. (2018). OS with Durvalumab after Chemoradiotherapy in Stage III NSCLC. N. Engl. J. Med..

[34] Faivre-Finn C., Vicente D., Kurata T., Planchard D., Paz-Ares L., Vansteenkiste J.F., Spigel D.R., Garassino M.C., Reck M., Senan S. (2021). Four-Year Survival with Durvalumab After Chemoradiotherapy in Stage III NSCLC-an Update from the PACIFIC Trial. J. Thorac. Oncol..

[35] Lee A., Duggan S., Deeks E.D. (2020). Cemiplimab: A Review in Advanced Cutaneous Squamous Cell Carcinoma. Drugs.

[36] Sezer A., Kilickap S., Gümüş M., Bondarenko I., Özgüroğlu M., Gogishvili M., Turk H.M., Cicin I., Bentsion D., Gladkov O. (2021). Cemiplimab monotherapy for first-line treatment of advanced non-small-cell lung cancer with PD-L1 of at least 50%: A multicentre, open-label, global, phase 3, randomised, controlled trial. Lancet.

[37] Camidge D.R., Doebele R.C., Kerr K.M. (2019). Comparing and contrasting predictive biomarkers for immunotherapy and targeted therapy of NSCLC. Nat. Rev. Clin. Oncol..

[38] Langer C.J., Gadgeel S.M., Borghaei H., Papadimitrakopoulou V.A., Patnaik A., Powell S.F., Gentzler R.D., Martins R.G., Stevenson J.P., Jalal S.I. (2016). Carboplatin and pemetrexed with or without pembrolizumab for advanced, non-squamous non-small-cell lung cancer: A randomised, phase 2 cohort of the open-label KEYNOTE-021 study. Lancet Oncol..

[39] Gadgeel S., Rodríguez-Abreu D., Speranza G., Esteban E., Felip E., Dómine M., Hui R., Hochmair M.J., Clingan P., Powell S.F. (2020). Updated Analysis From KEYNOTE-189: Pembrolizumab or Placebo Plus Pemetrexed and Platinum for Previously Untreated Metastatic Nonsquamous Non-Small-Cell Lung Cancer. J. Clin. Oncol..

[40] Paz-Ares L., Vicente D., Tafreshi A., Robinson A., Parra H.S., Mazières J., Hermes B., Cicin I., Medgyasszay B., Rodríguez-Cid J. (2020). A Randomized, Placebo-Controlled Trial of Pembrolizumab Plus Chemotherapy in Patients with Metastatic Squamous NSCLC: Protocol-Specified Final Analysis of KEYNOTE-407. J. Thorac. Oncol..

[41] West H., McCleod M., Hussein M., Morabito A., Rittmeyer A., Conter H.J., Kopp H.-G., Daniel D., McCune S., Mekhail T. (2019). Atezolizumab in combination with carboplatin plus nab-paclitaxel chemotherapy compared with chemotherapy alone as first-line treatment for metastatic non-squamous non-small-cell lung cancer (IMpower130): A multicentre, randomised, open-label, phase 3 trial. Lancet Oncol..

[42] Jotte R., Cappuzzo F., Vynnychenko I., Stroyakovskiy D., Rodríguez-Abreu D., Hussein M., Soo R., Conter H.J., Kozuki T., Huang K.-C. (2020). Atezolizumab in Combination with Carboplatin and Nab-Paclitaxel in Advanced Squamous NSCLC (IMpower131): Results from a Randomized Phase III Trial. J. Thorac. Oncol..

[43] Nishio M., Barlesi F., West H., Ball S., Bordoni R., Cobo M., Longeras P.D., Goldschmidt J., Novello S., Orlandi F. (2021). Atezolizumab Plus Chemotherapy for First-Line Treatment of Nonsquamous NSCLC: Results from the Randomized Phase 3 IMpower132 Trial. J. Thorac. Oncol..

[44] Rothschild S.I., Zippelius A., Eboulet E.I., Prince S.S., Betticher D., Bettini A., Früh M., Joerger M., Lardinois D., Gelpke H. (2021). SAKK 16/14: Durvalumab in Addition to Neoadjuvant Chemotherapy in Patients with Stage IIIA(N2) Non-Small-Cell Lung Cancer-A Multicenter Single-Arm Phase II Trial. J. Clin. Oncol..

[45] Yang Y., Wang Z., Fang J., Yu Q., Han B., Cang S., Chen G., Mei X., Yang Z., Ma R. (2020). Efficacy and Safety of Sintilimab Plus Pemetrexed and Platinum as First-Line Treatment for Locally Advanced or Metastatic Nonsquamous NSCLC: A Randomized, Double-Blind, Phase 3 Study (Oncology pRogram by InnovENT anti-PD-1-11). J. Thorac. Oncol..

[46] Banfill K., Giuliani M., Aznar M., Franks K., McWilliam A., Schmitt M., Sun F., Vozenin M.C., Finn C.F. (2021). Cardiac Toxicity of Thoracic Radiotherapy: Existing Evidence and Future Directions. J. Thorac. Oncol..

[47] Weichselbaum R.R., Liang H., Deng L., Fu Y.-X. (2017). Radiotherapy and immunotherapy: A beneficial liaison?. Nat. Rev. Clin. Oncol..

[48] Gameiro S., Ardiani A., Kwilas A., Hodge J.W. (2014). Radiation-induced survival responses promote immunogenic modulation to enhance immunotherapy in combinatorial regimens. Oncoimmunology.

[49] Formenti S.C., Rudqvist N.P., Golden E., Cooper B., Wennerberg E., Lhuillier C., Vanpouille-Box C., Friedman K., de Andrade L.C., Wucherpfennig K.W. (2018). Radiotherapy induces responses of lung cancer to CTLA-4 blockade. Nat. Med..

[50] Theelen W.S.M.E., Chen D., Verma V., Hobbs B.P., Peulen H.M.U., Aerts J.G.J.V., Bahce I., Niemeijer A.L.N., Chang J.Y., de Groot P.M. (2021). Pembrolizumab with or without radiotherapy for metastatic non-small-cell lung cancer: A pooled analysis of two randomised trials. Lancet Respir. Med..

[51] Altorki N.K., E McGraw T., Borczuk A.C., Saxena A., Port J.L., Stiles B.M., Lee B.E., Sanfilippo N.J., Scheff R.J., Pua B.B. (2021). Neoadjuvant durvalumab with or without stereotactic body radiotherapy in patients with early-stage non-small-cell lung cancer: A single-centre, randomised phase 2 trial. Lancet Oncol..

[52] Du S., Zhou L., Alexander G.S., Park K., Yang L., Wang N., Zaorsky N.G., Ma X., Wang Y., Dicker A.P. (2018). PD-1 Modulates Radiation-Induced Cardiac Toxicity through Cytotoxic T Lymphocytes. J. Thorac. Oncol..

[53] Parry R.V., Chemnitz J.M., Frauwirth K.A., Lanfranco A.R., Braunstein I., Kobayashi S.V., Linsley P.S., Thompson C.B., Riley J.L. (2005). CTLA-4 and PD-1 receptors inhibit T-cell activation by distinct mechanisms. Mol. Cell. Biol..

[54] Pardoll D.M. (2012). The blockade of immune checkpoints in cancer immunotherapy. Nat. Rev. Cancer.

[55] Mangsbo S.M., Sandin L.C., Anger K., Korman A.J., Loskog A., Tötterman T.H. (2010). Enhanced tumor eradication by combining CTLA-4 or PD-1 blockade with CpG therapy. J. Immunother..

[56] Hellmann M.D., Rizvi N.A., Goldman J.W., Gettinger S.N., Borghaei H., Brahmer J.R., Ready N.E., Gerber D.E., Chow L.Q., Juergens R.A. (2017). Nivolumab plus ipilimumab as first-line treatment for advanced non-small-cell lung cancer (CheckMate 012): Results of an open-label, phase 1, multicohort study. Lancet Oncol..

[57] Ready N., Hellmann M.D., Awad M.M., Otterson G.A., Gutierrez M., Gainor J.F., Borghaei H., Jolivet J., Horn L., Mates M. (2019). First-Line Nivolumab Plus Ipilimumab in Advanced Non-Small-Cell Lung Cancer (CheckMate 568): Outcomes by Programmed Death Ligand 1 and Tumor Mutational Burden as Biomarkers. J. Clin. Oncol..

[58] Hellmann M.D., Paz-Ares L., Bernabe Caro R., Zurawski B., Kim S.-W., Costa E.C., Park K., Alexandru A., Lupinacci L., Jimenez E.d.l.M. (2019). Nivolumab plus Ipilimumab in Advanced Non-Small-Cell Lung Cancer. N. Engl. J. Med..

[59] Paz-Ares L., Ciuleanu T.E., Cobo M., Schenker M., Zurawski B., Menezes J., Richardet E., Bennouna J., Felip E., Juan-Vidal O. (2021). First-line nivolumab plus ipilimumab combined with two cycles of chemotherapy in patients with non-small-cell lung cancer (CheckMate 9LA): An international, randomised, open-label, phase 3 trial. Lancet Oncol..

[60] Vellanki P.J., Mulkey F., Jaigirdar A.A., Rodriguez L., Wang Y., Xu Y., Zhao H., Liu J., Howe G., Wang J. (2021). FDA Approval Summary: Nivolumab with Ipilimumab and Chemotherapy for Metastatic Non-small Cell Lung Cancer, A Collaborative Project Orbis Review. Clin. Cancer Res..

[61] Cascone T., William W.N., Weissferdt A., Leung C.H., Lin H.Y., Pataer A., Godoy M.C.B., Carter B.W., Federico L., Reuben A. (2021). Neoadjuvant nivolumab or nivolumab plus ipilimumab in operable non-small cell lung cancer: The phase 2 randomized NEOSTAR trial. Nat. Med..

[62] Tanvetyanon T., Gray J.E., Antonia S.J. (2017). PD-1 checkpoint blockade alone or combined PD-1 and CTLA-4 blockade as immunotherapy for lung cancer?. Expert Opin. Biol. Ther..

[63] Hou J., Karin M., Sun B. (2021). Targeting cancer-promoting inflammation—Have anti-inflammatory therapies come of age?. Nat. Rev. Clin. Oncol..

[64] Chen N., Fang W., Zhan J., Hong S., Tang Y., Kang S., Zhang Y., He X., Zhou T., Qin T. (2015). Upregulation of PD-L1 by EGFR Activation Mediates the Immune Escape in EGFR-Driven NSCLC: Implication for Optional Immune Targeted Therapy for NSCLC Patients with EGFR Mutation. J. Thorac. Oncol..

[65] Zhou C., Wang Y., Zhao J., Chen G., Liu Z., Gu K., Huang M., He J., Chen J., Ma Z. (2021). Efficacy and Biomarker Analysis of Camrelizumab in Combination with Apatinib in Patients with Advanced Nonsquamous NSCLC Previously Treated with Chemotherapy. Clin. Cancer Res..

[66] Taylor M.H., Lee C.H., Makker V., Rasco D., Dutcus C.E., Wu J., Stepan D.E., Shumaker R.C., Motzer R.J. (2020). Phase IB/II Trial of Lenvatinib Plus Pembrolizumab in Patients with Advanced Renal Cell Carcinoma, Endometrial Cancer, and Other Selected Advanced Solid Tumors. J. Clin. Oncol..

[67] Yang J.C.-H., Shepherd F.A., Kim D.-W., Lee G.-W., Seok Lee J., Chang G.-C., Sook Lee S., Wei Y.-F., Gyoo Lee Y., Laus G. (2019). Osimertinib Plus Durvalumab versus Osimertinib Monotherapy in EGFR T790M-Positive NSCLC following Previous EGFR TKI Therapy: CAURAL Brief Report. J. Thorac. Oncol..

[68] Schoenfeld A., Arbour K., Rizvi H., Iqbal A., Gadgeel S., Girshman J., Kris M., Riely G., Yu H., Hellmann M. (2019). Severe immune-related adverse events are common with sequential PD-(L)1 blockade and osimertinib. Ann. Oncol..

[69] Liang H., Wang M. (2019). Prospect of immunotherapy combined with anti-angiogenic agents in patients with advanced non-small cell lung cancer. Cancer Manag. Res..

[70] Folkman J. (1971). Tumor angiogenesis: Therapeutic implications. N. Engl. J. Med..

[71] Tian L., Goldstein A., Wang H., Ching Lo H., Kim I.S., Welte T., Sheng K., Dobrolecki L.E., Zhang X., Putluri N. (2017). Mutual regulation of tumour vessel normalization and immunostimulatory reprogramming. Nature.

[72] Peske J.D., Woods A.B., Engelhard V.H. (2015). Control of CD8 T-Cell Infiltration into Tumors by Vasculature and Microenvironment. Adv. Cancer Res..

[73] Wada J., Suzuki H., Fuchino R., Yamasaki A., Nagai S., Yanai K., Koga K., Nakamura M., Tanaka M., Morisaki T. (2009). The contribution of vascular endothelial growth factor to the induction of regulatory T-cells in malignant effusions. Anticancer. Res..

[74] Yang Y., Li L., Jiang Z., Wang B., Pan Z. (2020). Anlotinib optimizes anti-tumor innate immunity to potentiate the therapeutic effect of PD-1 blockade in lung cancer. Cancer Immunol. Immunother..

[75] Han B., Li K., Zhao Y., Li B., Cheng Y., Zhou J., Lu Y., Shi Y., Wang Z., Jiang L. (2018). Anlotinib as a third-line therapy in patients with refractory advanced non-small-cell lung cancer: A multicentre, randomised phase II trial (ALTER0302). Br. J. Cancer.

[76] Han B., Li K., Wang Q., Zhang L., Shi J., Wang Z., Cheng Y., He J., Shi Y., Zhao Y. (2018). Effect of Anlotinib as a Third-Line or Further Treatment on OS of Patients with Advanced Non-Small Cell Lung Cancer: The ALTER 0303 Phase 3 Randomized Clinical Trial. JAMA Oncol..

[77] Chu T., Zhong R., Zhong H., Zhang B., Zhang W., Shi C., Qian J., Zhang Y., Chang Q., Zhang X. (2021). Phase 1b Study of Sintilimab Plus Anlotinib as First-line Therapy in Patients with Advanced NSCLC. J. Thorac. Oncol..

[78] Hegde P.S., Wallin J.J., Mancao C. (2018). Predictive markers of anti-VEGF and emerging role of angiogenesis inhibitors as immunotherapeutics. Semin. Cancer Biol..

[79] Chen D., Hurwitz H. (2018). Combinations of Bevacizumab with Cancer Immunotherapy. Cancer J..

[80] Socinski M.A., Jotte R.M., Cappuzzo F., Orlandi F., Stroyakovskiy D., Nogami N., Rodríguez-Abreu D., Moro-Sibilot D., Thomas C.A., Barlesi F. (2018). Atezolizumab for First-Line Treatment of Metastatic Nonsquamous NSCLC. N. Engl. J. Med..

[81] Reck M., Mok T.S.K., Nishio M., Jotte R.M., Cappuzzo F., Orlandi F., Stroyakovskiy D., Nogami N., Rodríguez-Abreu D., Moro-Sibilot D. (2019). Atezolizumab plus bevacizumab and chemotherapy in non-small-cell lung cancer (IMpower150): Key subgroup analyses of patients with EGFR mutations or baseline liver metastases in a randomised, open-label phase 3 trial. Lancet Respir. Med..

[82] Nogami N., Barlesi F., Socinski M.A., Reck M., Thomas C.A., Cappuzzo F., Mok T.S., Finley G., Aerts J.G., Orlandi F. (2021). IMpower150 Final Exploratory Analyses for Atezolizumab Plus Bevacizumab and Chemotherapy in Key NSCLC Patient Subgroups with EGFR Mutations or Metastases in the Liver or Brain. J. Thorac. Oncol..

[83] Socinski M.A., Nishio M., Jotte R.M., Cappuzzo F., Orlandi F., Stroyakovskiy D., Nogami N., Rodríguez-Abreu D., Moro-Sibilot D., Thomas C.A. (2021). IMpower150 Final OS Analyses for Atezolizumab Plus Bevacizumab and Chemotherapy in First-Line Metastatic Nonsquamous NSCLC. J. Thorac. Oncol..

[84] Duffy M.J., Crown J. (2019). Biomarkers for Predicting Response to Immunotherapy with Immune Checkpoint Inhibitors in Cancer Patients. Clin. Chem..

[85] Rizzo A., Ricci A.D. (2021). PD-L1, TMB, and other potential predictors of response to immunotherapy for hepatocellular carcinoma: How can they assist drug clinical trials?. Expert Opin. Investig. Drugs.

[86] Brody R., Zhang Y., Ballas M., Siddiqui M.K., Gupta P., Barker C., Midha A., Walker J. (2017). PD-L1 expression in advanced NSCLC: Insights into risk stratification and treatment selection from a systematic literature review. Lung Cancer.

[87] Horn L., Spigel D.R., Vokes E.E., Holgado E., Ready N., Steins M., Poddubskaya E., Borghaei H., Felip E., Paz-Ares L. (2017). Nivolumab Versus Docetaxel in Previously Treated Patients with Advanced Non-Small-Cell Lung Cancer: Two-Year Outcomes from Two Randomized, Open-Label, Phase III Trials (CheckMate 017 and CheckMate 057). J. Clin. Oncol..

[88] Herbst R.S., Soria J.-C., Kowanetz M., Fine G.D., Hamid O., Gordon M.S., Sosman J.A., McDermott D.F., Powderly J.D., Gettinger S.N. (2014). Predictive correlates of response to the anti-PD-L1 antibody MPDL3280A in cancer patients. Nature.

[89] Wimberly H., Brown J.R., Schalper K., Haack H., Silver M.R., Nixon C., Bossuyt V., Pusztai L., Lannin D.R., Rimm D.L. (2015). PD-L1 Expression Correlates with Tumor-Infiltrating Lymphocytes and Response to Neoadjuvant Chemotherapy in Breast Cancer. Cancer Immunol. Res..

[90] Patel S.P., Kurzrock R. (2015). PD-L1 Expression as a Predictive Biomarker in Cancer Immunotherapy. Mol. Cancer Ther..

[91] Park S., Choi Y., Kim J., Kho B., Park C., Oh I., Kim Y. (2020). Efficacy of immune checkpoint inhibitors according to PD-L1 tumor proportion scores in non-small cell lung cancer. Thorac. Cancer.

[92] Passiglia F., Commendatore O., Vitali M., Conca R. (2018). Immunotherapy in non-small-cell lung cancer: A bridge between research and clinical practice. Future Oncol..

[93] Chen J., Jiang C.C., Jin L., Zhang X.D. (2016). Regulation of PD-L1: A novel role of pro-survival signalling in cancer. Ann. Oncol..

[94] Rizvi N.A., Hellmann M.D., Snyder A., Kvistborg P., Makarov V., Havel J.J., Lee W., Yuan J., Wong P., Ho T.S. (2015). Cancer immunology. Mutational landscape determines sensitivity to PD-1 blockade in non-small cell lung cancer. Science.

[95] Yarchoan M., Hopkins A., Jaffee E.M. (2017). Tumor Mutational Burden and Response Rate to PD-1 Inhibition. N. Engl. J. Med..

[96] Hellmann M.D., Ciuleanu T.E., Pluzanski A., Lee J.S., Otterson G.A., Audigier-Valette C., Minenza E., Linardou H., Burgers S., Salman P. (2018). Nivolumab plus Ipilimumab in Lung Cancer with a High Tumor Mutational Burden. N. Engl. J. Med..

[97] Ettinger D.S., Wood D.E., Aggarwal C., Aisner D.L., Akerley W., Bauman J.R., Bharat A., Bruno D.S., Chang J.Y., Chirieac L.R. (2019). NCCN Guidelines Insights: Non-Small Cell Lung Cancer, Version 1.2020. J. Natl. Compr. Cancer Netw..

[98] Rizvi H., Sanchez-Vega F., La K., Chatila W., Jonsson P., Halpenny D., Plodkowski A., Long N., Sauter J.L., Rekhtman N. (2018). Molecular Determinants of Response to Anti-Programmed Cell Death (PD)-1 and Anti-Programmed Death-Ligand 1 (PD-L1) Blockade in Patients with Non-Small-Cell Lung Cancer Profiled with Targeted Next-Generation Sequencing. J. Clin. Oncol..

[99] Li T., Zhao L., Yang Y., Wang Y., Zhang Y., Guo J., Chen G., Qin P., Xu B., Ma B. (2021). T Cells Expanded from PD-1+ Peripheral Blood Lymphocytes Share More Clones with Paired Tumor-Infiltrating Lymphocytes. Cancer Res..

[100] Beatty G.L., Gladney W.L. (2015). Immune escape mechanisms as a guide for cancer immunotherapy. Clin. Cancer Res..

[101] Zitvogel L., Kroemer G. (2012). Targeting PD-1/PD-L1 interactions for cancer immunotherapy. Oncoimmunology.

[102] Farhood B., Najafi M., Mortezaee K. (2019). CD8^+^ cytotoxic T lymphocytes in cancer immunotherapy: A review. J. Cell Physiol..

[103] Masucci G.V., Cesano A., Hawtin R., Janetzki S., Zhang J., Kirsch I., Dobbin K.K., Alvarez J., Robbins P.B., Selvan S.R. (2016). Validation of biomarkers to predict response to immunotherapy in cancer: Volume I—Pre-analytical and analytical validation. J. Immunother. Cancer.

[104] Liu H., Zhang T., Ye J., Li H., Huang J., Li X., Wu B., Huang X., Hou J. (2012). Tumor-infiltrating lymphocytes predict response to chemotherapy in patients with advance non-small cell lung cancer. Cancer Immunol Immunother..

[105] Sanmamed M.F., Nie X., Desai S.S., Villaroel-Espindola F., Badri T., Zhao D., Kim A.W., Ji L., Zhang T., Quinlan E. (2021). A Burned-Out CD8^+^ T-cell Subset Expands in the Tumor Microenvironment and Curbs Cancer Immunotherapy. Cancer Discov..

[106] Creelan B., Wang C., Teer J., Toloza E., Mullinax J., Yao J., Koomen J., Kim S., Chiappori A., Saller J. (2020). Abstract CT056: Durable complete responses to adoptive cell transfer using tumor infiltrating lymphocytes (TIL) in non-small cell lung cancer (NSCLC): A phase I trial. Cancer Res..

[107] Oya Y., Kuroda H., Nakada T., Takahashi Y., Sakakura N., Hida T. (2020). Efficacy of Immune Checkpoint Inhibitor Monotherapy for Advanced Non-Small-Cell Lung Cancer with ALK Rearrangement. Int. J. Mol. Sci..

[108] Gainor J.F., Shaw A.T., Sequist L.V., Fu X., Azzoli C.G., Piotrowska Z., Huynh T.G., Zhao L., Fulton L., Schultz K.R. (2016). EGFR Mutations and ALK Rearrangements Are Associated with Low Response Rates to PD-1 Pathway Blockade in Non-Small Cell Lung Cancer: A Retrospective Analysis. Clin. Cancer Res..

[109] Dong Z.-Y., Zhang J.-T., Liu S.-Y., Su J., Zhang C., Xie Z., Zhou Q., Tu H.-Y., Xu C.-R., Yan L.-X. (2017). EGFR mutation correlates with uninflamed phenotype and weak immunogenicity, causing impaired response to PD-1 blockade in non-small cell lung cancer. Oncoimmunology.

[110] Kauffmann-Guerrero D., Tufman A., Kahnert K., Bollmann B.A., Reu S., Syunyaeva Z., Schneider C., Manapov F., Huber R.M., Golpon H. (2020). Response to Checkpoint Inhibition in Non-Small Cell Lung Cancer with Molecular Driver Alterations. Oncol. Res. Treat..

[111] Darvin P., Toor S.M., Sasidharan Nair V., Elkord E. (2018). Immune checkpoint inhibitors: Recent progress and potential biomarkers. Exp. Mol. Med..

[112] Buder-Bakhaya K., Hassel J.C. (2018). Biomarkers for Clinical Benefit of Immune Checkpoint Inhibitor Treatment-A Review from the Melanoma Perspective and Beyond. Front. Immunol..

[113] Augustus E., Zwaenepoel K., Siozopoulou V., Raskin J., Jordaens S., Baggerman G., Sorber L., Roeyen G., Peeters M., Pauwels P. (2021). Prognostic and Predictive Biomarkers in Non-Small Cell Lung Cancer Patients on Immunotherapy-The Role of Liquid Biopsy in Unraveling the Puzzle. Cancers.

[114] Schnipper L.E., Davidson N.E., Wollins D.S., Tyne C., Blayney D.W., Blum D., Dicker A.P., Ganz P.A., Hoverman J.R., Langdon R. (2015). American Society of Clinical Oncology Statement: A Conceptual Framework to Assess the Value of Cancer Treatment Options. J. Clin. Oncol..

[115] Büttner R., Gosney J.R., Skov B.G., Adam J., Motoi N., Bloom K.J., Dietel M., Longshore J.W., Lopez-Rios F., Penault-Llorca F. (2017). Programmed Death-Ligand 1 Immunohistochemistry Testing: A Review of Analytical Assays and Clinical Implementation in Non-Small-Cell Lung Cancer. J. Clin. Oncol..

[116] Velcheti V., Patwardhan P.D., Liu F.X., Chen X., Cao X., Burke T. (2018). Real-world PD-L1 testing and distribution of PD-L1 tumor expression by immunohistochemistry assay type among patients with metastatic non-small cell lung cancer in the United States. PLoS ONE.

[117] Lee W.S., Yang H., Chon H.J., Kim C. (2020). Combination of anti-angiogenic therapy and immune checkpoint blockade normalizes vascular-immune crosstalk to potentiate cancer immunity. Exp. Mol. Med..

[118] Brahmer J.R., Lacchetti C., Schneider B.J., Atkins M.B., Brassil K.J., Caterino J.M., Chau I., Ernstoff M.S., Gardner J.M., Ginex P. (2018). Management of Immune-Related Adverse Events in Patients Treated with Immune Checkpoint Inhibitor Therapy: American Society of Clinical Oncology Clinical Practice Guideline. J. Clin. Oncol..

[119] Naidoo J., Wang X., Woo K.M., Iyriboz T., Halpenny D., Cunningham J., Chaft J.E., Segal N.H., Callahan M.K., Lesokhin A.M. (2017). Pneumonitis in Patients Treated with Anti-Programmed Death-1/Programmed Death Ligand 1 Therapy. J. Clin. Oncol..

[120] Hamanishi J., Mandai M., Matsumura N., Abiko K., Baba T., Konishi I. (2016). PD-1/PD-L1 blockade in cancer treatment: Perspectives and issues. Int. J. Clin. Oncol..

[121] Suresh K., Naidoo J., Lin C.T., Danoff S. (2018). Immune Checkpoint Immunotherapy for Non-Small Cell Lung Cancer: Benefits and Pulmonary Toxicities. Chest.

[122] De Velasco G., Je Y., Bossé D., Awad M.M., Ott P.A., Moreira R.B., Schutz F., Bellmunt J., Sonpavde G.P., Hodi F.S. (2017). Comprehensive Meta-analysis of Key Immune-Related Adverse Events from CTLA-4 and PD-1/PD-L1 Inhibitors in Cancer Patients. Cancer Immunol. Res..

[123] Khunger M., Rakshit S., Pasupuleti V., Hernandez A.V., Mazzone P., Stevenson J., Pennell N.A., Velcheti V. (2017). Incidence of Pneumonitis with Use of Programmed Death 1 and Programmed Death-Ligand 1 Inhibitors in Non-Small Cell Lung Cancer: A Systematic Review and Meta-Analysis of Trials. Chest.

[124] Suresh K., Voong K.R., Shankar B., Forde P.M., Ettinger D.S., Marrone K.A., Kelly R.J., Hann C.L., Levy B., Feliciano J.L. (2018). Pneumonitis in Non-Small Cell Lung Cancer Patients Receiving Immune Checkpoint Immunotherapy: Incidence and Risk Factors. J. Thorac. Oncol..

[125] Naidoo J., Page D.B., Li B.T., Connell L.C., Schindler K., Lacouture M.E., Postow M.A., Wolchok J.D. (2015). Toxicities of the anti-PD-1 and anti-PD-L1 immune checkpoint antibodies. Ann. Oncol..

[126] Moaaz M., Youssry S., Elfatatry A., El Rahman M.A. (2019). Th17/Treg cells imbalance and their related cytokines (IL-17, IL-10 and TGF-β) in children with autism spectrum disorder. J. Neuroimmunol..

[127] Ciciola P., Cascetta P., Bianco C., Formisano L., Bianco R. (2020). Combining Immune Checkpoint Inhibitors with Anti-Angiogenic Agents. J. Clin. Med..

[128] Robert C. (2018). Is earlier better for melanoma checkpoint blockade?. Nat. Med..

